# A genome-wide association study of seed protein and oil content in soybean

**DOI:** 10.1186/1471-2164-15-1

**Published:** 2014-01-02

**Authors:** Eun-Young Hwang, Qijian Song, Gaofeng Jia, James E Specht, David L Hyten, Jose Costa, Perry B Cregan

**Affiliations:** 1 Department of Plant Science and Landscape Architecture, University of Maryland, College Park, MD 20742, USA; 2 USDA, Agricultural Research Service, Soybean Genomics and Improvement Lab, Beltsville, MD 20705, USA; 3 Agronomy & Horticulture Department, University of Nebraska, Lincoln, NE 68583, USA; 4 Present address: DuPont Pioneer, 8305 NW 62nd Ave., PO Box 7060, Johnston, IA 50131, USA; 5 Present address: USDA-ARS, Crop Production and Protection, GWCC-BLTSVL, Beltsville, MD 20705, USA

**Keywords:** GWAS, *Glycine max*, Seed protein and oil content, Single nucleotide polymorphism, Linkage disequilibrium

## Abstract

**Background:**

Association analysis is an alternative to conventional family-based methods to detect the location of gene(s) or quantitative trait loci (QTL) and provides relatively high resolution in terms of defining the genome position of a gene or QTL. Seed protein and oil concentration are quantitative traits which are determined by the interaction among many genes with small to moderate genetic effects and their interaction with the environment. In this study, a genome-wide association study (GWAS) was performed to identify quantitative trait loci (QTL) controlling seed protein and oil concentration in 298 soybean germplasm accessions exhibiting a wide range of seed protein and oil content.

**Results:**

A total of 55,159 single nucleotide polymorphisms (SNPs) were genotyped using various methods including Illumina Infinium and GoldenGate assays and 31,954 markers with minor allele frequency >0.10 were used to estimate linkage disequilibrium (LD) in heterochromatic and euchromatic regions. In euchromatic regions, the mean LD (*r*^*2*^) rapidly declined to 0.2 within 360 Kbp, whereas the mean LD declined to 0.2 at 9,600 Kbp in heterochromatic regions. The GWAS results identified 40 SNPs in 17 different genomic regions significantly associated with seed protein. Of these, the five SNPs with the highest associations and seven adjacent SNPs were located in the 27.6-30.0 Mbp region of Gm20. A major seed protein QTL has been previously mapped to the same location and potential candidate genes have recently been identified in this region. The GWAS results also detected 25 SNPs in 13 different genomic regions associated with seed oil. Of these markers, seven SNPs had a significant association with both protein and oil.

**Conclusions:**

This research indicated that GWAS not only identified most of the previously reported QTL controlling seed protein and oil, but also resulted in narrower genomic regions than the regions reported as containing these QTL. The narrower GWAS-defined genome regions will allow more precise marker-assisted allele selection and will expedite positional cloning of the causal gene(s).

## Background

Association studies provide an alternative to conventional family-based methods for detecting the genomic location of genes or quantitative trait loci (QTL). While both rely on the correlation between DNA marker alleles and the phenotypic expression of a trait of interest, association studies can provide relatively higher resolution in terms of defining the genomic position of a gene or QTL, since it can be applied to naturally occurring populations such as human populations or germplasm collections. The important difference between the degree of QTL detection possible with these natural populations and that possible with progeny derived from crosses between two individuals is the level of linkage disequilibrium (LD).

LD is the non-random association of alleles at different loci in a population. The detection of genes or QTL depends on the level of LD between a causal mutation and physically linked markers. The higher the degree of association between marker alleles and the phenotypic variants, the greater the likelihood that the phenotypic causal mutation is physically linked to the marker. A confounding factor that influences the success of association study is the presence of population structure. Population structure is the result of allele frequency differences between different populations that arise as a result of population history. Factors such as selection, migration, local adaptation, geographical isolation, or genetic drift can result in population structure. It is well known that population structure can cause spurious associations between markers and the trait under study [1], because of allele frequency differences between subpopulations in a population, rather than genuine genetic associations with the trait of interest.

Owing to the lack of sufficient numbers of DNA markers, early association studies in plant species used the candidate gene approach to identify specific single nucleotide polymorphisms (SNPs) or genes controlling a phenotypic trait of interest such as, flowering time [2], endosperm color [3] and kernel starch biosynthesis [4] in maize (*Zea mays* ssp. mays), enzymatic discoloration in potato (*Solanum tuberosum* L*.*) [5], flowering time in sorghum (*Sorghum bicolor* L. Moench) [6], and aluminum tolerance in triticale (X Triticosecale Wittmack) [7]. However, the availability of high throughput DNA sequencing and genotyping technologies has provided a platform for conducting genome-wide association studies (GWAS). Such studies have verified the location of loci associated with frost tolerance in barley (*Hordeum vulgare* L.) [8]; leaf architecture [9]; southern leaf blight [10], and waterlogging tolerance [11] in maize (*Zea mays* L.); agronomic traits in rice (*Oryza sativa* L.) [12]; flowering time [13] and defense metabolites [14] in Arabidopsis (*Arabidopsis thaliana* L. Heynh) and plant architecture and flowering time in sunflower [15]. In soybean (*Glycine max* L. Merr.), a GWAS was performed to detect genes/markers associated with iron deficiency chlorosis [16] and chlorophyll and chlorophyll fluorescence parameters [17].

Seed protein and oil content in soybean are quantitatively inherited traits determined by the interaction of a number of genes subject to genotype × environment interactions. Many seed protein and oil QTL have been reported in a number of studies over the past two decades (SoyBase, the USDA, ARS Soybean Genetics and Genomics Database). These QTL, identified using linkage analysis of populations derived from crosses of two parents with contrasting seed protein and oil concentration, have been detected in many different genomic regions throughout all 20 chromosomes. Several of these QTL have been identified three or more times at identical or very similar chromosomal positions in different populations, which suggests that these QTL are not likely false positives. Because these regions likely contain a gene or genes with relatively large genetic effects on seed protein and oil content, their re-identification in a GWAS targeted at seed protein and oil would provide a measure of the success of GWAS in an independent test of its ability to detect the presence of seed protein and oil QTL.

In the research reported here, the degree of LD was assessed in 298 soybean germplasm accessions obtained from the USDA Soybean Germplasm Collection. Seed protein and oil concentration were measured in seeds harvested from plants grown in replicated field trials at two locations. The resulting seed composition data were used to evaluate the GWAS approach, and ultimately to provide an assessment of the likely success of GWAS when it is used to detect the QTL controlling the two well-known soybean quantitative traits–seed protein and oil content.

## Results

### Linkage disequilibrium in euchromatic and heterochromatic genome regions

**Figure 1:**
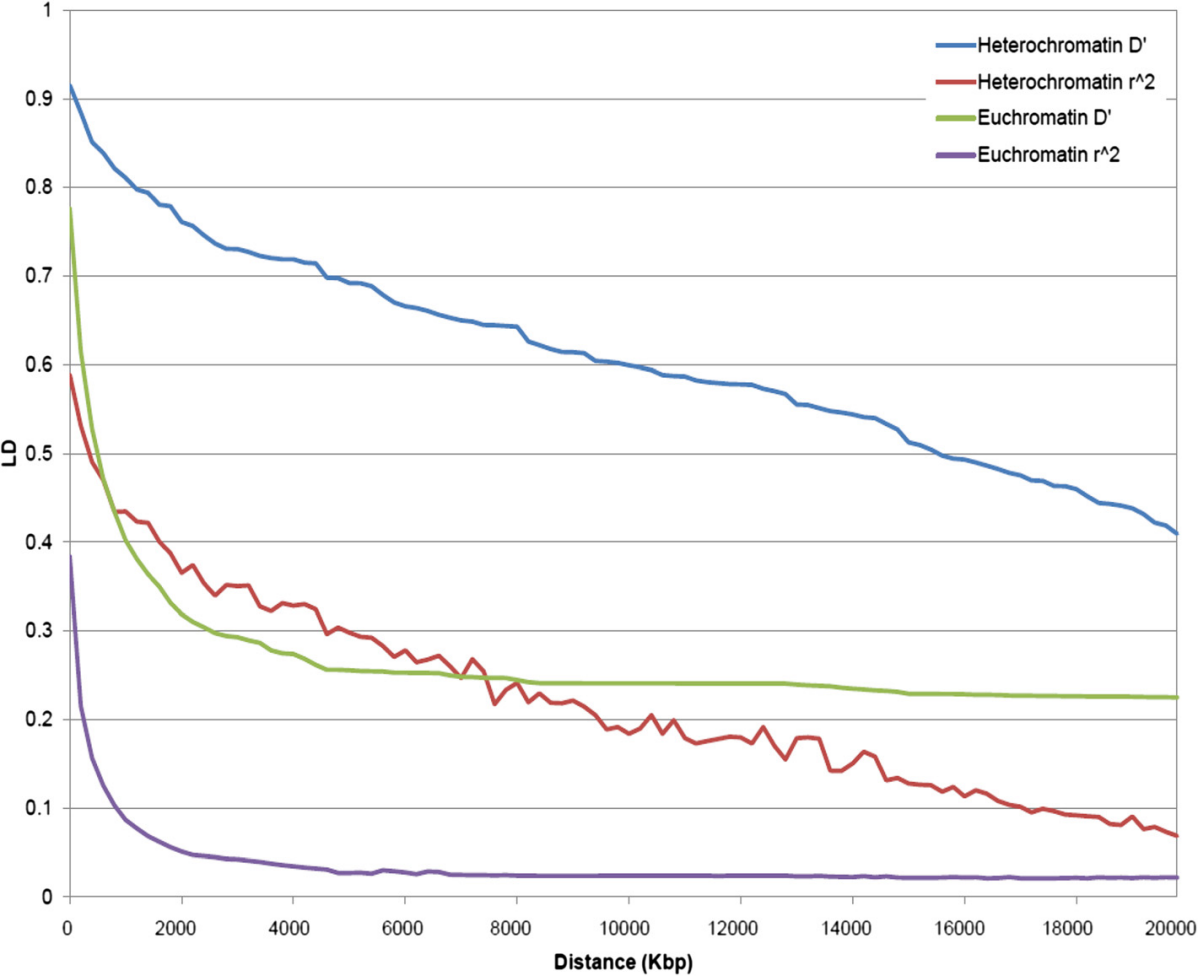
**The mean level of LD in heterochromatic and euchromatic chromosome regions.** The mean LD was estimated using all pairs of loci located within 20 Mbp of physical distance. The X-axis indicates the distance between marker pairs and the Y-axis indicates LD level. The green and purple lines respectively denote mean *D’* and mean *r*^*2*^ in euchromatic regions, and the blue and red lines respectively denote mean *D’* and mean *r*^*2*^ in heterochromatic regions.

A total of 31,954 SNPs distributed across the soybean genome with minor allele frequency >0.10 and missing data of less than 25% was used for the estimation of LD level in the 298 soybean germplasm accessions. These SNP markers spanned 950.1 Mbp, which represents approximately 86.4% of the 1.1 Gbp soybean genome, resulting in an average SNP density of 1 SNP every 17.0 Kbp in euchromatic regions and 1 SNP every 100.1 Kbp in heterochromatic regions. In euchromatic regions, the mean level of LD measured by *D’* declined very rapidly to 0.5 within 600 Kbp, and mean LD as measured by *r*^*2*^ dropped sharply to 0.2 at 360 Kbp (Figure 1). However, in heterochromatic regions, the mean value of *D’* remained greater than 0.5 until about 15,000 Kbp and mean LD as measured by *r*^*2*^ did not decline to 0.2 until about 9,600 Kbp (Figure 1).

### Population structure

A total of 42,368 SNPs positioned on the 20 soybean chromosomes was used to estimate the most likely number (*K*) of subgroups in the 298 germplasm accessions. The most likely *K* value was *K* = 17. The accessions within seven subgroups originated from a single Asian country and the other nine subgroups were comprised of a mix of accessions from different countries. In terms of maturity group, eight subgroups consisted of accessions with the same maturity group, whereas the other eight subgroups were a mix of accessions of different maturity groups.

### Seed protein and oil phenotypes

**Figure 2:**
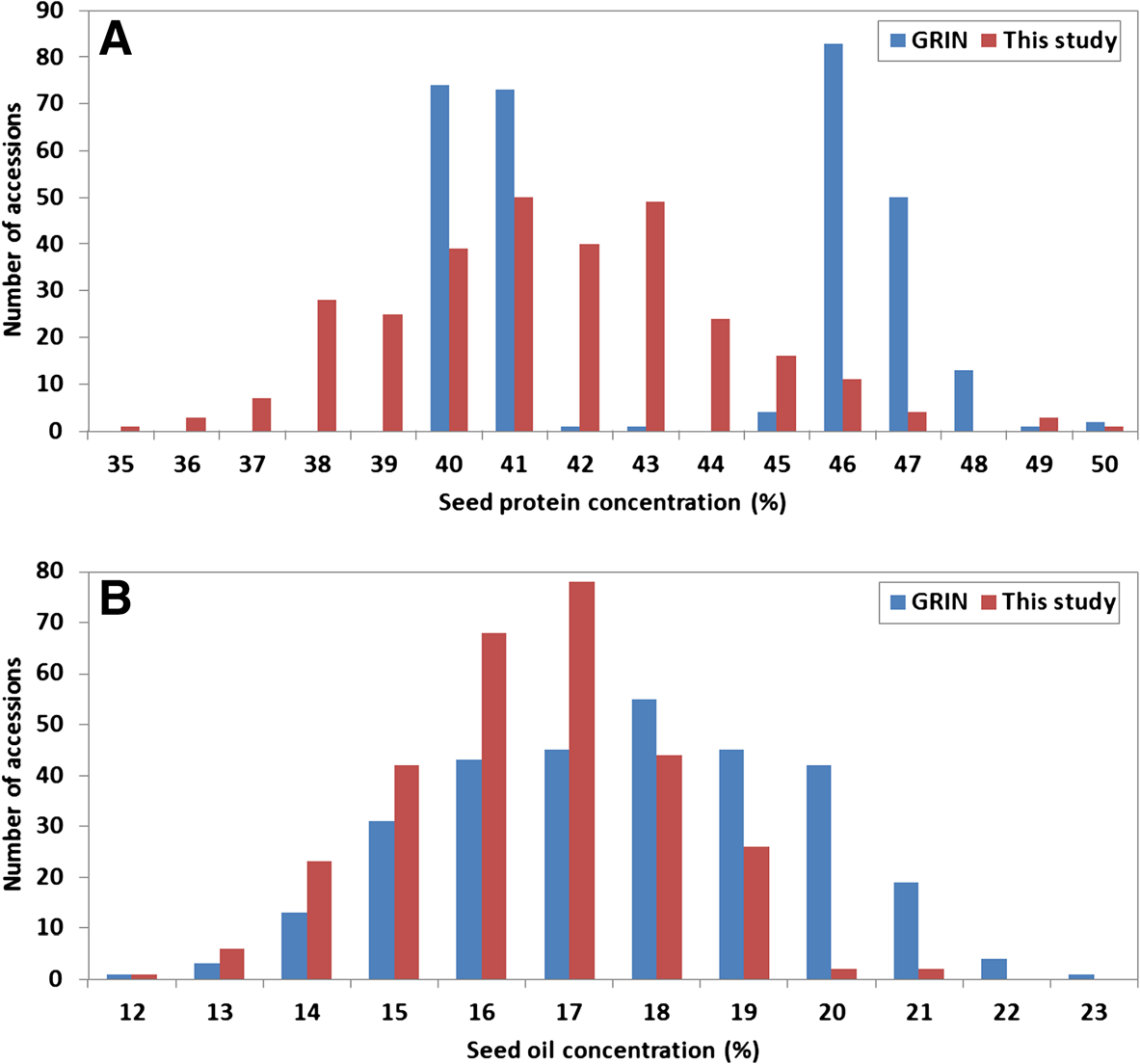
**Seed protein and oil concentration in the GRIN database *vs*. that determined in this study.** Seed protein **(A)** and seed oil **(B)** concentrations of the soybean germplasm accessions, respectively, reported in the GRIN database *vs*. the percentage determined in this study from seed harvested from two-replicate trials conducted at two locations (Beltsville, MD; and Lincoln, NE) in 2003. Blue bars are data from the GRIN database and red bars are data from this study.

The germplasm accessions were selected based upon data in the USDA Germplasm Resources Information Network (GRIN) database (http://www.ars-grin.gov/), to represent two distinct groups–one group with normal seed protein content (40 to 43%) and the other with high seed protein content (46 to 51%). However, seed protein content measured in seeds grown in replicated hill-plot trials at Beltsville, MD and Lincoln, NE showed continuous variation with a range from 35 to 50% (Figure 2A). Experimental error was low in each trial, given the coefficient of variation (CV) values of 5.77% in the MD experiment and 5.99% in the NE experiment, with an overall CV of 5.67% in the 2-location analysis. The analysis of variance indicated that the accessions differed significantly (P < 0.0001) in seed protein content, and no significant interaction between accessions and locations was detected (Table 1). The correlation coefficient (r) of seed protein concentration between the MD and NE experiments was quite high, r = 0.98 (P < 0.0001). The correlation of the accession mean seed protein measured in the NE and MD experiments with the GRIN accession protein value was moderate, r = 0.62 (P < 0.0001) and r = 0.61 (P < 0.0001), respectively. The seed oil content of the accessions displayed a pattern similar to the seed protein content relative to the GRIN oil values (Figure 2B). The analysis of variance for seed oil revealed that accessions were significantly different (P < 0.0001) and as was the case with protein, there was no significant accession × location interaction for oil (Table 2). The correlation of accession seed oil content between the two locations was high, r = 0.95 (P < 0.0001), but was only moderately high, r = 0.77 (P < 0.0001) and r = 0.78 (P < 0.0001), between the GRIN accession values and those measured at the NE and MD locations, respectively. The correlation between mean seed protein and oil contents in the MD experiment and in the NE experiment were r = -0.64 and r = -0.66, respectively. The heritability of seed protein and seed oil concentrations was 77.9% and 78.33%, respectively. Such heritability values are higher than would be typical in breeding studies.

**Table 1:** Analysis of variance of seed protein content

**Source**	**DF**	**SS**	**MS**	**F value**	**Pr > F**
Accession	297	6874.21	23.15	77.6	<0.0001
Location	1	6.95	6.95	23.2	0.0389
Accession × location	297	99.06	0.3	0.185	1
Replications (locations)	2	4.59	2.29	1.413	0.2442
Error	518	840.5	1.62		

Analysis of variance of harvested seed of *G. max* accessions from plants grown in 2003 in replicated hill plot trials conducted in fields located in Beltsville, MD and near Lincoln, NE.

**Table 2:** Analysis of variance of seed oil content

**Source**	**DF**	**SS**	**MS**	**F value**	**Pr > F**
Accession	290	2414.49	8.33	14.88	<0.0001
Location	1	0.69	0.69	1.23	0.3667
Accession × location	290	66.06	0.23	0.411	1
Replications (locations)	2	3.49	1.74	3.12	0.045
Error	520	291	0.56		

Analysis of variance of harvested seed of *G. max* accessions from plants grown in 2003 in replicated hill plot trials conducted in fields located in Beltsville, MD and near Lincoln, NE.

### Genome-wide association study for the genes controlling seed protein and oil concentration

**Figure 3:**
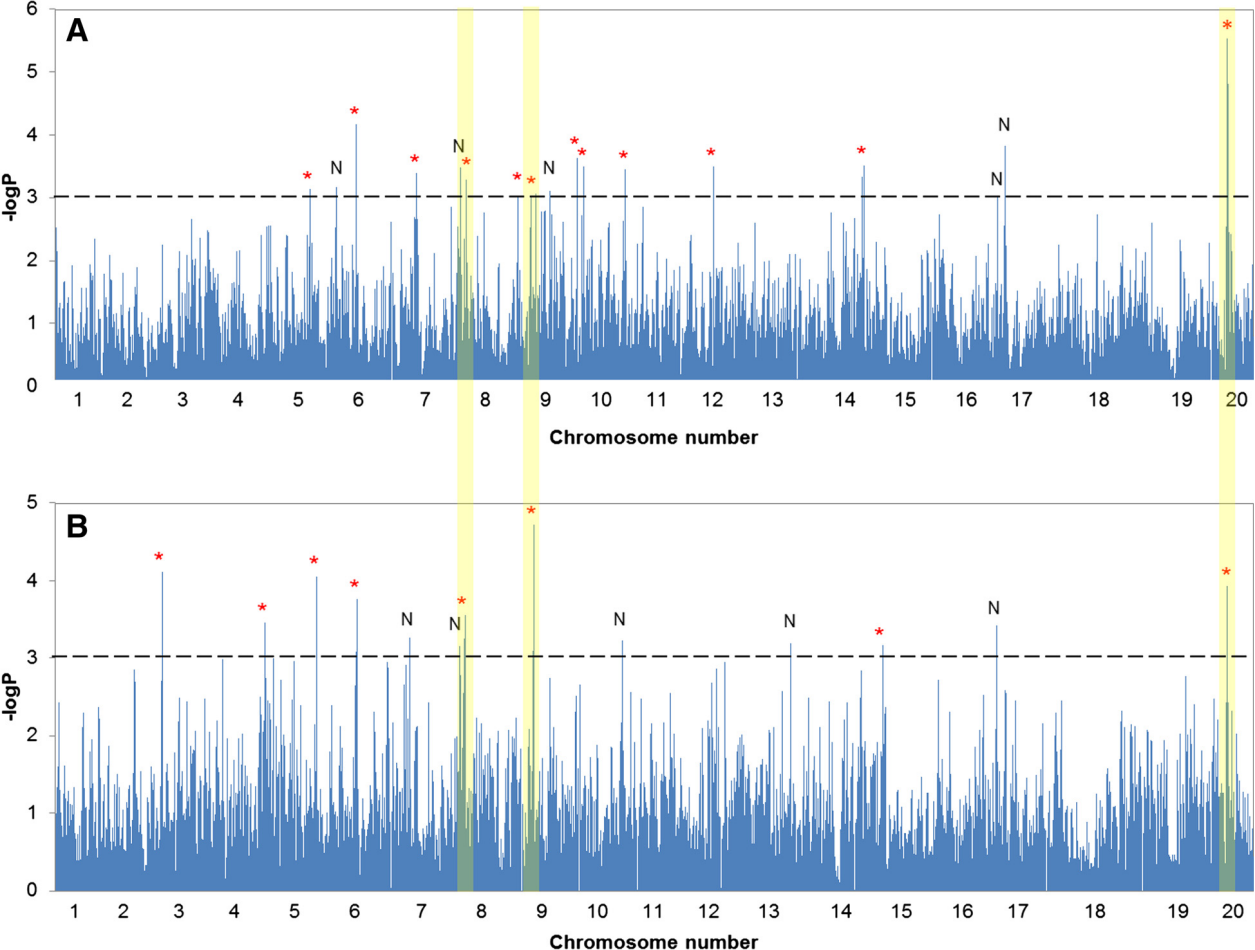
**GWAS for seed protein and oil concentration.** Manhattan plots depicting the extent of the association of 31,954 SNPs, dispersed as shown over the 20 soybean chromosomes, with **(A)** mean seed protein content and **(B)** mean seed oil content of the soybean accessions, respectively. The-log P value is a measure of the degree to which a SNP is associated with the trait. SNP spikes topped by a red asterisk (*) denote a genomic region aligning with the location of a previously reported QTL, whereas those topped with the letter N denote that the region may harbor a heretofore unreported QTL. The vertical yellow bars spanning both graphs denote specific markers or a few markers in close proximity that exhibit significant association with both seed protein and oil content.

**Figure 4:**
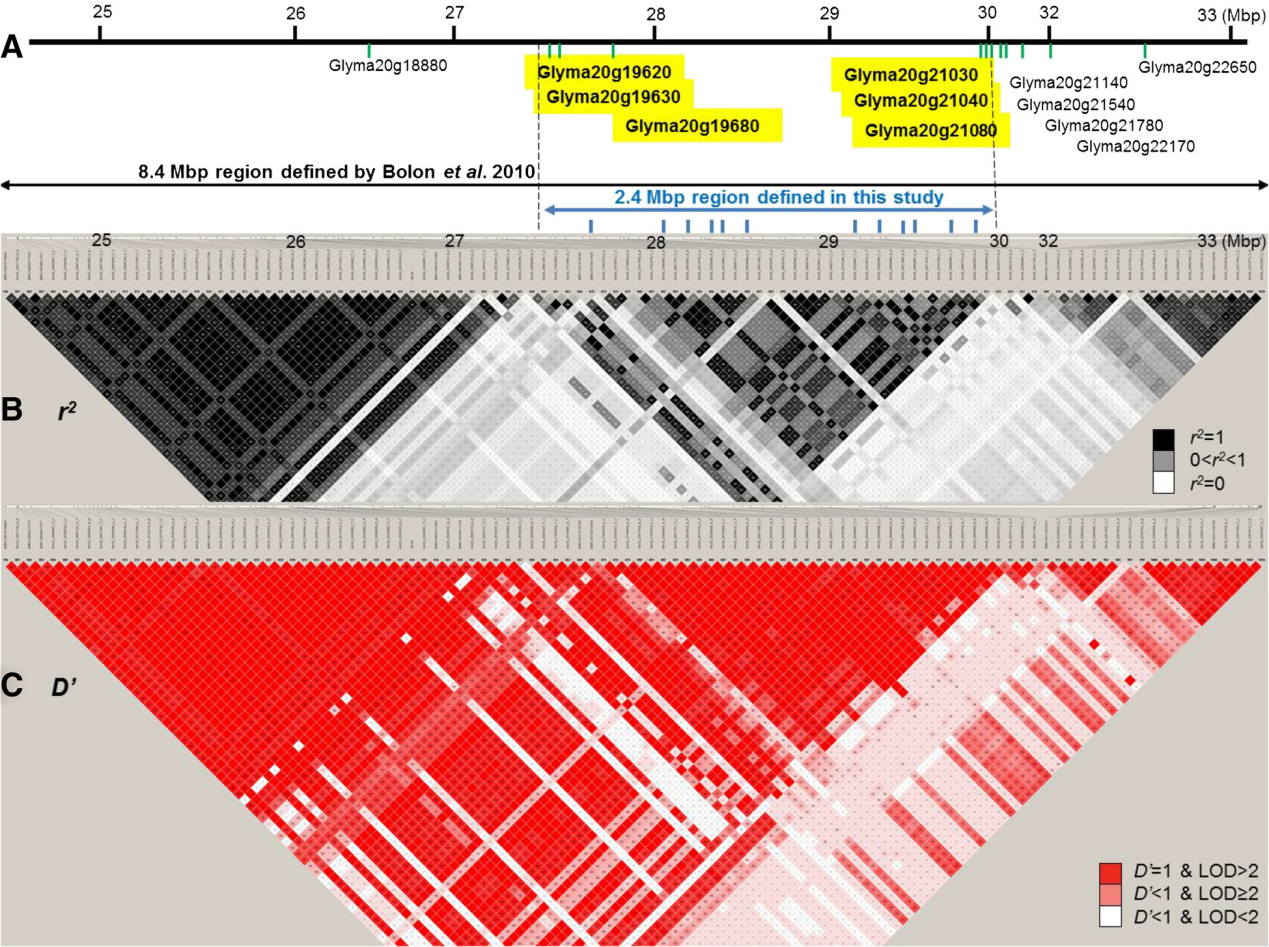
**The candidate region of the major seed protein QTL on Gm20.** Plots depicting an 8.4 Mbp region of Gm20 that is suspected of harboring a candidate gene responsible for a major pleiotropic soybean seed protein/oil QTL. **Panel A)** depicts the 12 potential candidate genes (Glyma names) and the physical positions of the genes indicated with green vertical lines as reported by Bolon *et al*. [36] in the 8.4 Mbp region that, based upon the GWAS findings in this report, can be narrowed to a 2.4 Mbp region that harbors only six (yellow-highlighted) of the 12 candidate genes. The physical positions of the 12 SNPs associated with protein content are indicated with blue vertical lines. **Panel B)** depicts the extent of LD in this region based on *r*^*2*^ (in black) and **Panel C)** depicts LD based on the *D’* (in red). Note: The original 12 gene models whose positions are shown in the 8.4 Mbp region in the top panel were differentially expressed in a pair of high *vs*. low seed protein soybean near-isogenic lines–see Bolon *et al*. [36] for additional details.

A GWAS was performed with the mixed linear model (MLM) which greatly reduced false positive rates as shown in quantile-quantile plots (Additional file 1). The results of the genome-wide association study for seed protein content are presented in Figure 3A. A significant association (-log P > 3) with seed protein concentration was observed for 40 SNPs located in 17 different genomic regions in 10 of the 20 chromosomes (Additional file 2). Nine of the 17 regions had more than two markers with significant association and the physical distance between markers ranged from 1,786 to 1,723,908 bp. More than a quarter (i.e., 12) of these 40 SNPs had physical positions in the 27.9-30.0 Mbp segment of Gm20, all of which were in complete LD as indicated by *D’* (Figure 4). Of the 40 SNPs, five SNPs in the 27.9-30.0 Mbp segment of Gm20 exhibited the highest association with seed protein content. This is not surprising given the fact that this is a heterochromatic region with very extensive LD, while the 28 other markers with significant association to protein content were located in euchromatic regions where the mean level of LD declines very rapidly. A significant association with seed oil content was detected for 25 SNPs, and these were physically located in 13 regions on 12 of the 20 chromosomes (Figure 3B and Additional file 3) where seven regions included more than two markers with the distance between markers ranging from 5,108 to 470,370 bp. All of these markers were located in euchromatic regions, except the four markers located on Gm20. The SNP showing the highest association with oil (i.e., a-log P value of 4.71) was located at the 4.92 Mbp position on Gm09. There were seven markers from three regions on Gm08, Gm09, and Gm20 associated with both protein and oil seed content (Table 3). At six of these seven marker loci there was a negative relationship between the protein effect versus that on oil i.e., the allele associated with increased protein content was always associated with decreased oil content or vice versa. The aforementioned SNP at the 4.92 Mbp position on Gm09 was the exception–one allele was associated with increased protein and oil content and the alternative allele with both lower protein and oil content.

**Table 3:** SNP loci associated with both seed protein and oil content

**Chr**	**SNP ID**	**-logP**		**Mean protein content (%) associated with SNP allele**				**Mean oil content (%) associated with SNP allele**				**Difference**		**Protein *vs*. oil relationship**^***a***^
		**Protein**	**Oil**	**G**	**A**	**T**	**C**	**G**	**A**	**T**	**C**	**Protein**	**Oil**	
8	BARC_1.01_Gm08_9683120_C_T	3.22	3.25			42.35	40.08			16.78	18.35	2.27	1.57	Negative
8	BARC_1.01_Gm08_9741542_T_C	3.28	3.55			39.95	42.37			18.43	16.76	2.42	1.66	Negative
9	BARC_1.01_Gm09_4921120_C_T	3.06	4.71			41.61	42.15			16.41	17.06	0.55	0.65	Positive
20	BARC_1.01_Gm20_29395999_T_C	5.52	3.93			41.46	44.32			17.34	15.59	2.86	1.75	Negative
20	BARC_1.01_Gm20_29512680_A_G	4.96	3.69	44.22	41.46			15.65	17.34			2.76	1.69	Negative
20	BARC_1.01_Gm20_29594697_A_G	5.52	3.93	44.32	41.46			15.59	17.34			2.86	1.75	Negative
20	BARC_1.01_Gm20_29983050_A_G	4.82	3.03	44.32	41.50			15.65	17.30			2.82	1.65	Negative

The mean protein and oil content of the genotypes with the alternative SNP alleles, the difference in protein and oil content between the genotypes with the alternative alleles and the direction (negative or positive) of the protein *vs*. oil effects associated with the alleles at each locus.

^*a*^A negative relationship indicates that the allele associated with greater protein content was associated with reduced oil content and vice versa. A positive relationship indicates that the allele associated with greater protein content was also associated with greater oil content and the alternative allele was associated with both reduced protein and oil content.

## Discussion

Soybean germplasm accessions used in this study that include landraces collected in China, Korea, and Japan over a 60 year period represent an excellent reservoir of genetic variation for the application of a GWAS. It is assumed that these accessions possess a diversity of alleles. Such diversity can be examined (and possibly exploited) for soybean genetic improvement by identifying alleles that would be useful in achieving that genetic improvement. GWAS offers one approach for accomplishing that objective.

### Linkage disequilibrium

An important consideration in the application of genome-wide association for gene discovery is the extent of LD. Because of obvious differences in the extent of LD between different chromosomal regions, we elected to define LD separately for the euchromatic and heterochromatic regions of the 20 soybean chromosomes. The reliability of the approach used in the present study, i.e., to define the heterochromatic region of each chromosome as the region between the two inflection points of the cumulative genetic distance plotted against the cumulative physical distance, was previously examined in rice. In that species, the heterochromatic regions defined by suppressed recombination rate was in agreement with that identified by the conventional DAPI staining method [18]. The plot of genetic distance on physical distance clearly indicated a higher recombination per physical distance in euchromatic regions *vs*. heterochromatic regions. Approximately five times greater recombination per unit of physical distance occurs in soybean euchromatic versus heterochromatic regions. In the grass species, sorghum (*Sorghum bicolor* (L.) Moench), the recombination rate in euchromatic regions was approximately 34 times higher than that in heterochromatic regions [19]. In fact, 97-98% of the recombination occurred in the euchromatin of this species [20]. The difference in recombination rate between the heterochromatic regions and the euchromatic regions explains the difference in the extent of LD observed in the two chromosomal regions. The different levels of LD as indicated by *r*^*2*^ between different species and different chromosome regions within species can be explained by a combination of population histories including mating system, mutation rate, founding effects, the magnitude of selection, admixture, and genetic drift. The large difference in the LD level in euchromatic and heterochromatic regions, as shown in the present study, supports our contention that the density of markers required for GWAS should be adjusted to take into account the changing ratio, along the length of a given chromosome, of recombination to genetic distance. In euchromatic regions with high recombination to physical distance, a high marker density will be required, whereas in heterochromatic regions, a lower marker density is acceptable. Of course, until a whole genome sequence and a densely populated genetic map become available, it is not possible to precisely define the ratio of genetic to physical distance across the genome of a species to which a GWAS is being conducted.

### Association analysis for seed protein and oil content

The challenge for association studies in crop plants is the identification of genes associated with quantitatively inherited agriculturally important traits. Currently, many seed protein and oil QTL have been reported at many positions across the 20 soybean chromosomes/linkage groups in numerous studies (SoyBase, http://www.soybase.org). The genome positions where QTL were reported a number of times in studies using different sources of high seed protein and oil germplasm should be good candidates for the validation of associations detected via GWAS. All the previously reported seed protein and oil QTL listed in SoyBase were identified using linkage analysis and thus, the causal gene(s) could be located a substantial distance (in cM) from the markers which were reported to be genetically linked to the QTL. Therefore, the exact position of the QTL determined by linkage analysis could not be precisely determined. However, with the release of Version 4.0 of the soybean genetic map [21], which was produced from a JoinMap 3.0 analysis of five soybean mapping populations that collectively segregated for over 5,000 markers, it is possible to more narrowly define the regions to which previously reported protein and oil QTL had been mapped. Using this information, it is possible with some confidence to relate the physical position of the reported QTL from different studies with the SNP positions identified by GWAS in the present study. In that regard, we were able to align the 17 genome regions associated with seed protein identified in this study with previously reported seed protein QTL locations, and based on that alignment, determined that QTL had previously been reported in 12 of the 17 regions (Figure 3A and Additional file 2). Notably, the seed protein QTL on Gm20 has been reported seven times, the QTL at the 14.7 Mbp position on Gm06 six times, the regions located on Gm05, Gm07, and Gm15 four times, the region on Gm09 three times, and the regions at 43.9 Mbp on Gm08, 1.4 Kbp on Gm10, and on Gm12 have been reported in two previous studies. Of the 13 regions associated with seed oil content, the regions on Gm05, Gm06, and Gm20 had been previously reported more than three times and the regions on Gm02 and Gm04, the region at 9.9 Mbp on Gm09, and the region on Gm15 have been reported twice. In addition, there were QTL that have been reported in previous studies which could not be found in this study. One reason could be that since the accessions used in this study were from the maturity groups II, III and IV, QTL identified in accessions from other maturity groups are not present in the germplasm accessions used in this study. For example, we did not find QTL reported in studies using very early or late maturity groups such as three QTL identified in populations derived from maturity group 00 × 00 [22], three QTL from maturity group 0 × 0 [23,24], nine QTL from maturity group VI × VI [25], and three QTL from maturity group 0 × I [26,27]. A second reason may be that many protein and oil QTL have been identified in a number of different populations, however, if a QTL is controlled by a rare allele present only in a specific accession used in creating a QTL mapping population, it could not be detected in a GWAS such as is reported here. The inability of GWAS to detect rare alleles occurring in one or a few members of a population under study is well documented [28,29].

The main differences between the findings of an association study versus linkage analysis are firstly, the ability to detect a range of genes controlling the phenotype under study rather than just those segregating in a given mating of two parents and secondly, the increased resolution resulting from historical recombination rather than the limited recombination in the progeny of a biparental population. The QTL identified on Gm20 provides an example of the increased resolution of GWAS. This seed protein QTL has been reported seven times in different *G. soja* × *G. max* and *G. max* × *G. max* populations with *R*^*2*^ values ranging from 0.15 to 0.65 [22,30-35]. This QTL on Gm20 has been recognized as a candidate region for possibly cloning the causal gene(s) controlling seed protein content. Bolon *et al*. [36] analyzed gene expression of developing seeds in a pair of near isogenic lines (NILs) contrasting in seed protein concentration and differing for the region of Gm20 containing this major seed protein QTL. Differential transcript accumulation in the developing seeds of the NILs was detected for 12 candidate genes. The region defined by Bolon *et al*. [36] in which these genes reside spans approximately 8.4 Mbp from 24.5 to 32.9 Mbp on Gm20. Based on the results of the LD estimation in the current study, we verified that the region defined by Bolon *et al*. [36] possessed two large LD blocks, as determined by *r*^*2*^, each spanning regions of approximately 3 Mbp, one from 24.5 to 27.6 Mbp and the other from 27.6 to 30.0 Mbp (Figure 4). All the SNPs showing significant association with seed protein concentration were located in the 27.6 to 30.0 Mbp region. Thus, our GWAS results support a narrowing of the candidate gene region of this major seed protein QTL on Gm20 to about 2.4 Mbp versus the previously defined region of 8.4 Mbp [36]. Only six of the 12 genes identified by Bolon *et al*. [36] were located within the GWAS-defined 2.4 Mbp region (Figure 4). The gene candidates that remain include Hsp22.5 (Glyma20g19680), a putative ammonium transporter AMT1 (Glyma20g21030), an ATP synthase D chain (Glyma20g21080) (Figure 4) and three genes with limited similarity to known genes. We can assume that one or more of these genes may likely be the causative gene(s) associated with soybean seed protein content. This high resolution mapping demonstrates the capacity of GWAS to utilize historical recombination to increase resolution [37].

The GWAS for seed oil concentration also successfully defined 13 chromosome regions with high resolution, of which eight regions corresponded with previously reported oil QTL. Thus, in the case of both seed protein and oil content, five chromosome regions were identified at which no previously reported QTL had been detected. These regions may be of particular interest to soybean breeders and geneticists as sources of genetic variation to alter soybean seed protein and oils levels.

It has been recognized that there is a negative correlation between seed size and seed protein concentration [32,34], but more importantly, an even stronger negative correlation between seed oil and seed protein content [22,38-41]. *G. soja* has an extremely small seed size and much higher seed protein concentration (>50%) [42,43] than *G. max*. The average seed protein content in most *G. max* germplasm accessions is approximately 42.1% [44]. The reasonable explanation could be that selection had been imposed for larger seed types during domestication by ancient soybean farmers which had a concurrent genetic consequence of lower seed protein concentration. The lower seed protein concentration resulting from selection would also simultaneously lead to the increase in seed oil concentration. The possible cause for this would be either very tight genetic linkage of the genes controlling seed protein and oil concentration or that both traits are controlled by the same gene(s) [30]. The latter is more likely, given that high seed protein and the low seed oil almost always co-segregate and given the fact that many attempts to separate low seed oil from high seed protein (or the inverse) have been unsuccessful [30,32]. The results of GWAS from the current study were not in complete agreement with this assertion given results for the SNP at the 4.92 Mbp position on Gm09 at which one allele was associated with both higher protein and oil content and the alternative allele with lower protein and oil content. This is a QTL that should be of interest to those breeding for seed constituents.

One suggested virtue of association studies is the ability to take advantage of existing phenotypic data. However, there are also obstacles to performing association studies using existing phenotypic data, especially in the case of quantitative traits such as soybean seed protein and oil content. The seed protein and oil data obtained from GRIN were derived from field evaluations of the USDA Soybean Germplasm Collection conducted over the past 50 years at various locations and the evaluations included accessions from one or more maturity groups. The seed protein and oil data for the accessions used in the current study were not from a single field trail or set of trials in which all of the 298 accessions were included. Rather, the data were from a number of different trials grown in different environments and years. A quantitative trait defined in different studies and determined in a range of different environments and years is likely to produce different accession values that are confounded by these non-genetic effects, and it is, of course, the accession means that are used in GWAS. Such may be the case of the seed protein and oil concentration from the GRIN database used in this study (Figure 2A and B). There was only a correlation of r = 0.61 for seed protein concentration and 0.78 for seed oil concentration between the replicated field data from the current study *vs*. the data reported in the GRIN. We have a high level of confidence in the data obtained in the current study given the low coefficient of variation in the two replicated field experiments at two locations and the high correlation of the seed protein and oil values between the two locations. Thus, it is at least a possibility that the advantage often suggested for association study, i.e., the availability of large amounts of existing phenotypic data may, in some cases, not be the advantage that is often assumed.

## Conclusions

In this study, we performed a GWAS to detect genome regions controlling the quantitative traits, seed protein and oil content, using 42,368 SNP markers in a genetically diverse set of the 298 soybean germplasm accessions. Despite the relatively low level of LD and complex population structure, we were able to successfully identify many of the previously reported QTL associated with soybean seed protein and oil, and we also were able to further narrow the size of the genome region in which those QTL were likely to be located. The chromosome regions defined in this study can be used for further analysis to identify the causal gene(s) as well as to identify DNA markers that can be used in selection to alter soybean seed protein and oil in a predictable manner. The SNP data used in this study will soon be available through SoyBase (http://soybase.org/) for more than 19,000 *G. max* and *G. soja* germplasm accessions in the USDA Soybean Germplasm Collection based upon the analysis of the entire USDA Soybean Germplasm Collection with the Illumina Infinium SoySNP50K BeadChip described by Song *et al*. [45]. These genotypic data should be a useful resource for the detection of agriculturally important genes/QTL using association analysis.

## Methods

### Plant materials

Soybean germplasm accessions were selected based upon seed protein content (% on a dry weight basis) as reported in the GRIN (Germplasm Resources Information Network, U.S. Department of Agriculture, Agricultural Research Service, http://www.ars-grin.gov/npgs/index.html) database (Additional file 4). A total of 298 accessions were identified so as to create two groups–a *case* group including 151 accessions with high seed protein values ranging from 46 to 51%, and a *control* group including 147 accessions with more typical seed protein values ranging from 40 to 43%. The selection of accessions for the two groups was constrained to ensure balance between the groups in terms of geographical origin in the Far East (China, Korea, Japan), maturity group (II, III & IV), and other known phenotypic and morphological traits. Such balancing was done in an attempt to mitigate differences in population structure between the two groups. Genomic DNA of all accessions was extracted from bulked young leaf tissue grown in the greenhouse using the CTAB method [46].

### Field trials and measurement of protein and oil in the harvested seed

Field tests were conducted using a randomized complete block design with two replicates of hillplots grown at Beltsville, MD and two replicates grown at Lincoln, NE in 2003. For an analysis of total seed nitrogen, the seeds were dried for one day at 60°C and ground with a coffee grinder. The powdered grain was stored in an air-tight polyethylene bag and weighed in small tin capsules (LECO, St. Joseph, MI) to a targeted powder weight of approximately 0.2 g. The percentage of total nitrogen in the grain powder was determined using a LECO CHN 2000 analyzer (LECO, St. Joseph, MI) [47]. The seed protein percentage was calculated by multiplying the total nitrogen percentage by 6.25. The seed oil percentage was determined with approximately 10 g of seed using a Maran pulsed NMR (Resonance Instruments, Witney, Oxfordshire, UK), followed by the field induction decay-spin echo procedure [48]. Protein and oil concentrations were expressed on a% dry weight basis.

### Genotyping

A total of 55,159 single nucleotide polymorphisms (SNPs) were genotyped in the 298 accessions, with 52,041 analyzed using an Illumina Infinium assay [45] and 3,072 SNPs analyzed using the Illumina GoldenGate assay following the protocol described by Fan *et al*. [49] and Hyten *et al*. [50]. In addition, there were 46 SNP markers genotyped by direct sequencing and a single-base-extension method using the Luminex flow cytometer as described by Choi *et al*. [51]. A total of 1,363 SNPs were analyzed using both Illumina GoldenGate and Illumina Infinium assays. After eliminating redundant SNPs, non-polymorphic SNPs and SNPs with >25% missing data, a total of 42,368 SNPs (Additional file 5) remained. The physical positions of these markers in the soybean genome were determined using the whole genome assembly of Williams 82 soybean (Glyma 1.01) at the U.S. Department of Energy, Joint Genome Institute, Walnut Creek, CA (http://www.phytozome.net/soybean).

### Linkage disequilibrium estimation

For the estimation of the level of LD, a total of 31,954 loci with minor allele frequency >0.10 and the number of missing data points less than 25% was used. Heterozygous alleles were treated as missing data. Only physically linked markers located within 20 Mbp distances were used for LD estimation. Haploview 4.2 [52] was used to make all pair-wise comparisons of the alleles to calculate *r*^*2*^ (the squared allele frequency correlation between two loci), and to compute *D’* (standardized disequilibrium coefficient) [53]. In addition, LD (*D’* and *r*^*2*^) was estimated separately for euchromatic and heterochromatic regions. The euchromatin and the heterochromatin regions of each of the 20 chromosomes were determined as follows: The physical positions of 3,321 SNP and 862 simple sequence repeat (SSR) markers mapped in the soybean genome [21,51,54-56] were determined by BLAST analysis of the SNP and SSR-containing source sequences to the soybean whole genome sequence using the standalone Megablast software as previously described [57]. The cumulative genetic distances (cM) [21] were plotted against their cumulative physical distance (Mbp) to determine the base pair/centiMorgan relationship via the common SSR and SNP loci positions on the genetic linkage map and their physical position in the genome sequence along each chromosome. The region between the two inflection points of the cumulative genetic distance against cumulative physical distance on the plot was defined as the heterochromatin and the regions on each chromosome flanking the inflection points were defined as the euchromatin [45]. In order to provide an assessment of the difference in the extent of LD between euchromatic and heterochromatic regions, LD was calculated by a pairwise comparison of physically linked heterochromatic SNPs, and then two separate LD calculations were made for all pairs of markers from the two flanking euchromatic regions on each chromosome. The mean value of LD was estimated by calculating the mean LD of SNP pairs at distances of 0-200 Kbp, 200-400 Kbp, etc. to 19,800-20,000 Kbp in euchromatic and heterochromatic regions.

### Statistical analysis

An analysis of variance was conducted to obtain the variance components which were used to calculate the heritability of seed protein and oil content. The variances of location, replications within locations, accessions, and the accession × location interaction were determined using the PROC GLM procedure of the Statistical Analysis System (SAS institute, Inc., Cary, NC). Genetic and environmental variances were extracted from the variance component estimates based on the expected mean squares. For the estimation of the heritability of seed protein and seed oil concentration, replications and locations were considered to be random effects.

The heritability of seed protein and oil concentration was defined as

$$h_{B}^{2}=\frac{\sigma_{g}^{2}}{\sigma_{g}^{2}+\sigma_{e}^{2}},$$

where $\sigma_{g}^{2}$ is the genetic variance among accessions, and $\sigma_{e}^{2}$ is the environmental variance which results from error and the accession × location interaction.

To obtain the matrix of population structure, a total of 42,368 SNPs were analyzed in the 298 germplasm accessions using the Admixture program v. 1.22 [58]. The 10-fold cross-validation procedure was performed with 25 random seeding replications for *K* values from 2 to 30. The minimum mean standard error was when *K* = 17. The kinship coefficient matrix that explained the most probable identity by state of each allele between individuals was estimated with the TASSEL program [59]. For a genome-wide association study, we compared the false positive rate using the general linear model (GLM), the mixed linear model (MLM), and the compressed MLM of the TASSEL program [59]. The MLM was as good as the compressed MLM and greatly reduced the false positive rate versus the GLM. For this study, the value of 0.001 was used as a Type I error significance threshold P value. As a verification of the genome regions identified in this study we compared the genomic locations of previously reported seed protein and oil QTL with the physical positions of the markers showing significant associations in this study.

## Abbreviations

GWAS: Genome-wide association study; SNPs: Single nucleotide polymorphisms; LD: Linkage disequilibrium; QTL: Quantitative trait locus; GRIN: Germplasm resources information network; SSR: Simple sequence repeat; cM: Centimorgan; CV: Coefficient of variation; K: Structure matrix; RFLP: Restriction fragment length polymorphism; NILs: Near isogenic lines; GLM: General linear model; MLM: And checks and the three experiments in mixed linear model.

## Competing interests

The authors declare that they have no competing interests.

## Authors’ contributions

PBC designed and supervised the research; EYH analyzed the data, QS carried out the statistical analysis; GJ performed the Infinium assay; JES conducted the field trial to evaluate the seed protein and oil content; JC, EYH and PBC wrote the manuscript. All authors read and approved the manuscript.

## Supplementary Material

Additional file 1 **Quantile-quantile plots for seed protein and oil content.** Quantile-quantile plots of the general linear model (GLM) for seed protein **(A)** and oil **(B)**, the mixed linear model (MLM) for seed protein **(C)** and oil **(D)**, and the compressed MLM for seed protein **(E)** and oil **(F)**. Click here for file

Additional file 2 **SNP markers associated with seed protein content QTL.** Significantly associated markers (based on a -logP > 3.0) are numbered consecutively in the first column. The second column reports whether the QTL has been previously reported and the third column reports whether a marker(s) in the regions is also associated with seed oil content. Click here for file

Additional file 3 **SNP markers associated with seed oil content QTL.** Significantly associated markers (based on a-logP > 3.0) are numbered consecutively in the first column. The second column reports whether the QTL has been previously reported and the third column reports whether a marker(s) in the region is also associated with seed protein content. Click here for file

Additional file 4 **Soybean germplasm accessions analyzed in this study.** Soybean germplasm accessions [with Plant Introduction (PI) numbers] analyzed in this study and information including country of origin and phenotypic data reported in GRIN. The rightmost four columns display the seed protein and oil values reported in GRIN and those measured in the present study. Click here for file

Additional file 5 **The SNP allele present at 42,369 SNP loci analyzed in the 298 soybean germplasm accessions.** Data includes the SNP Locus Name, the NCBI Assay ID (ss#), the chromosome number (Chr) on which the SNP is located in the Williams 82 soybean whole genome sequence (Glyma 1.01 assembly, DOE, JGI http://www.phytozome.net/soybean) and the physical position in basepairs in the Glyma 1.01 assembly. Click here for file
