# Supplementary material for: A genome-wide association study of seed protein and oil content in soybean
Source: BMC Genomics. 2014 Jan 2;15:1. doi: 10.1186/1471-2164-15-1 (PMC3890527; doi:10.1186/1471-2164-15-1)
Supplement: Additional file 1 — Quantile-quantile plots for seed protein and oil content. Quantile-quantile plots of the general linear model (GLM) for seed protein (A) and oil (B), the mixed linear model (MLM) for seed protein (C) and oil (D), and the compressed MLM for seed protein (E) and oil (F). [file 1471-2164-15-1-S1.doc]

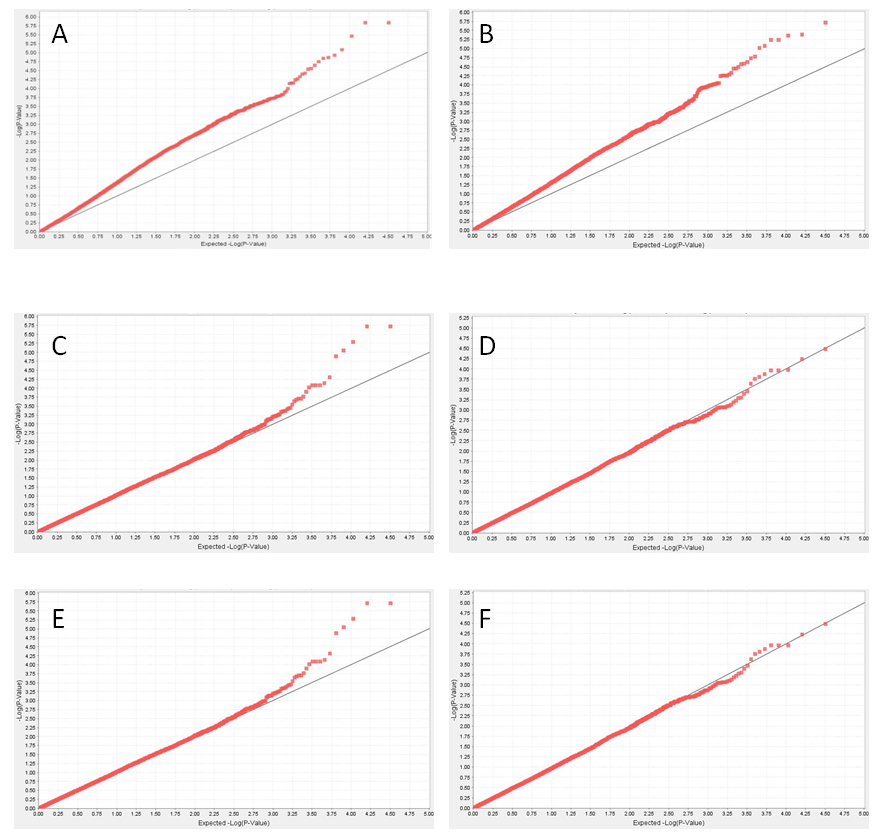


**Additional file 1 - Quantile-quantile plots for seed protein and oil content.**

Quantile-quantile plots of the general linear model (GLM) for seed protein (A) and oil (B), the mixed linear model (MLM) for seed protein (C) and oil (D), and the compressed MLM for seed protein (E) and oil (F).
