# Supplementary material for: A genome-wide association study of seed protein and oil content in soybean
Source: BMC Genomics. 2014 Jan 2;15:1. doi: 10.1186/1471-2164-15-1 (PMC3890527; doi:10.1186/1471-2164-15-1)
Supplement: Additional file 2 — SNP markers associated with seed protein content QTL. Significantly associated markers (based on a -logP > 3.0) are numbered consecutively in the first column. The second column reports whether the QTL has been previously reported and the third column reports whether a marker(s) in the regions is also associated with seed oil content. [file 1471-2164-15-1-S2.doc]

**Additional file 2 - SNP Markers associated with seed protein content QTL.**

Significantly associated markers (based on a -logP>3.0) are numbered consecutively in the first column. The second column reports whether the QTL has been previously reported and the third column reports whether a marker(s) in the regions is also associated with seed oil content.

| Protein QTL | Linkage analysis^a^ | Associated trait^b^ | Gm | LG | Marker name | Physical position (bp) | -logP | p |
| --- | --- | --- | --- | --- | --- | --- | --- | --- |
| 1 | Known | Oil | 5 | A1 | BARC_1.01_Gm05_35860291_T_C | 35,860,291 | 3.13 | 7.38E-04 |
|  |  |  | 5 | A1 | BARC_1.01_Gm05_35860939_C_T | 35,860,939 | 3.10 | 7.94E-04 |
|  |  |  | 5 | A1 | BARC_1.01_Gm05_36028909_A_G | 36,028,909 | 3.08 | 8.33E-04 |
| 2 | New |  | 6 | C2 | BARC_1.01_Gm06_1837479_C_T | 1,837,479 | 3.17 | 6.76E-04 |
| 3 | Known | Oil | 6 | C2 | BARC_1.01_Gm06_14737902_A_G | 14,737,902 | 4.17 | 6.82E-05 |
|  |  |  | 6 | C2 | BARC_1.01_Gm06_14743864_C_A | 14,743,864 | 3.67 | 2.12E-04 |
| 4 | Known | Oil | 7 | M | BARC_1.01_Gm07_9512225_A_G | 9,512,225 | 3.39 | 4.04E-04 |
| 5 | New | Oil | 8 | A2 | BARC_1.01_Gm08_3804209_G_A | 3,804,209 | 3.10 | 7.88E-04 |
|  |  |  | 8 | A2 | BARC_1.01_Gm08_3820272_A_C | 3,820,272 | 3.48 | 3.30E-04 |
| 6 | Known | Oil | 8 | A2 | BARC_1.01_Gm08_9683120_C_T | 9,683,120 | 3.22 | 6.00E-04 |
|  |  |  | 8 | A2 | BARC_1.01_Gm08_9741542_T_C | 9,741,542 | 3.28 | 5.25E-04 |
| 7 | Known |  | 8 | A2 | BARC_1.01_Gm08_43905753_A_C | 43,905,753 | 3.03 | 9.31E-04 |
| 8 | Known | Oil | 9 | K | BARC_1.01_Gm09_3197212_A_C | 3,197,212 | 3.01 | 9.74E-04 |
|  |  |  | 9 | K | BARC_1.01_Gm09_4921120_C_T | 4,921,120 | 3.06 | 8.64E-04 |
| 9 | New |  | 9 | K | BARC_1.01_Gm09_26254006_C_T | 26,254,006 | 3.11 | 7.84E-04 |
| 10 | Known |  | 9 | K | BARC_1.01_Gm09_45344827_A_G | 45,344,827 | 3.63 | 2.36E-04 |
| 11 | Known |  | 10 | O | BARC_1.01_Gm10_1397410_C_A | 1,397,410 | 3.50 | 3.17E-04 |
|  |  |  | 10 | O | BARC_1.01_Gm10_1399196_C_T | 1,399,196 | 3.42 | 3.83E-04 |
| 12 | Known | Oil | 10 | O | BARC_1.01_Gm10_44553009_T_C | 44,553,009 | 3.45 | 3.57E-04 |
| 13 | Known |  | 12 | H | BARC_1.01_Gm12_35509887_A_G | 35,509,887 | 3.08 | 8.39E-04 |
|  |  |  | 12 | H | BARC_1.01_Gm12_35520893_T_C | 35,520,893 | 3.50 | 3.16E-04 |
| 14 | Known |  | 15 | E | BARC_1.01_Gm15_3050845_G_T | 3,050,845 | 3.33 | 4.62E-04 |
|  |  |  | 15 | E | BARC_1.01_Gm15_3155475_C_A | 3,155,475 | 3.09 | 8.10E-04 |
|  |  |  | 15 | E | BARC_1.01_Gm15_3163558_A_G | 3,163,558 | 3.24 | 5.73E-04 |
|  |  |  | 15 | E | BARC-042349-08247 | 3,966,854 | 3.51 | 3.09E-04 |
|  |  |  | 15 | E | BARC-016533-02084 | 4,026,372 | 3.52 | 3.05E-04 |
| 15 | New | Oil | 17 | D2 | BARC_1.01_Gm17_4875540_T_C | 4,875,540 | 3.03 | 9.35E-04 |
| 16 | New |  | 17 | D2 | BARC-012687-00367 | 8,988,012 | 3.83 | 1.49E-04 |
| 17 | Known | Oil | 20 | I | BARC_1.01_Gm20_27884457_A_G | 27,884,457 | 3.18 | 6.67E-04 |
|  |  |  | 20 | I | BARC_1.01_Gm20_28017701_T_C | 28,017,701 | 3.00 | 9.92E-04 |
|  |  |  | 20 | I | BARC_1.01_Gm20_28070832_A_G | 28,070,832 | 3.18 | 6.67E-04 |
|  |  |  | 20 | I | BARC_1.01_Gm20_28208490_A_G | 28,208,490 | 3.02 | 9.55E-04 |
|  |  |  | 20 | I | BARC_1.01_Gm20_28276064_T_C | 28,276,064 | 3.18 | 6.67E-04 |
|  |  |  | 20 | I | BARC_1.01_Gm20_28439588_A_G | 28,439,588 | 3.24 | 5.72E-04 |
|  |  |  | 20 | I | BARC_1.01_Gm20_29208847_T_C | 29,208,847 | 3.11 | 7.69E-04 |
|  |  |  | 20 | I | BARC_1.01_Gm20_29395999_T_C | 29,395,999 | 5.52 | 2.99E-06 |
|  |  |  | 20 | I | BARC_1.01_Gm20_29512680_A_G | 29,512,680 | 4.96 | 1.10E-05 |
|  |  |  | 20 | I | BARC_1.01_Gm20_29594697_A_G | 29,594,697 | 5.52 | 2.99E-06 |
|  |  |  | 20 | I | BARC_1.01_Gm20_29865868_C_T | 29,865,868 | 5.01 | 9.77E-06 |
|  |  |  | 20 | I | BARC_1.01_Gm20_29983050_A_G | 29,983,050 | 4.82 | 1.52E-05 |

^a^  “Known” indicates that the chromosome region corresponded to a previously reported seed protein QTL and “New” indicates a new association identified in this study.

^b^ The word “Oil” indicates either the marker or a marker located in close proximity is also associated with seed oil concentration.
