# Supplementary material for: A genome-wide association study of seed protein and oil content in soybean
Source: BMC Genomics. 2014 Jan 2;15:1. doi: 10.1186/1471-2164-15-1 (PMC3890527; doi:10.1186/1471-2164-15-1)
Supplement: Additional file 3 — SNP markers associated with seed oil content QTL. Significantly associated markers (based on a-logP > 3.0) are numbered consecutively in the first column. The second column reports whether the QTL has been previously reported and the third column reports whether a marker(s) in the region is also associated with seed protein content. [file 1471-2164-15-1-S3.doc]

Additional file 3. SNP markers associated with seed oil content QTL detected (based on a -logP > 3.0) are numbered consecutively in the first column. The second column reports whether the QTL has been previously reported and the third column reports whether a marker(s) in the region is also associated with seed protein content

| Oil QTL | Linkage analysis^a^ | Associated trait^b^ | Gm | LG | Marker name | Physical position (bp) | -logP | p |
| --- | --- | --- | --- | --- | --- | --- | --- | --- |
| 1 | Known |  | 2 | D1b | BARC_1.01_Gm02_45758374_G_A | 45,758,374 | 3.06 | 8.75E-04 |
|  |  |  | 2 | D1b | BARC_1.01_Gm02_45805567_A_G | 45,805,567 | 3.89 | 1.30E-04 |
|  |  |  | 2 | D1b | BARC_1.01_Gm02_45811985_G_T | 45,811,985 | 4.11 | 7.69E-05 |
| 2 | Known |  | 4 | C1 | BARC-021803-04215 | 42,716,317 | 3.46 | 3.46E-04 |
| 3 | Known | Protein | 5 | A1 | BARC_1.01_Gm05_38543317_T_C | 38,543,317 | 3.98 | 1.04E-04 |
|  |  |  | 5 | A1 | BARC_1.01_Gm05_38569452_T_G | 38,569,452 | 4.05 | 8.87E-05 |
| 4 | Known | Protein | 6 | C2 | BARC_1.01_Gm06_15179985_G_A | 15,179,985 | 3.08 | 8.27E-04 |
|  |  |  | 6 | C2 | BARC_1.01_Gm06_15640480_T_C | 15,640,480 | 3.76 | 1.73E-04 |
| 5 | New | Protein | 7 | M | BARC_1.01_Gm07_8087301_C_T | 8,087,301 | 3.26 | 5.53E-04 |
| 6 | New | Protein | 8 | A2 | BARC_1.01_Gm08_4873149_C_T | 4,873,149 | 3.15 | 7.12E-04 |
| 7 | Known | Protein | 8 | A2 | BARC_1.01_Gm08_9683120_C_T | 9,683,120 | 3.25 | 5.58E-04 |
|  |  |  | 8 | A2 | BARC_1.01_Gm08_9689100_C_T | 9,689,100 | 3.25 | 5.58E-04 |
|  |  |  | 8 | A2 | BARC_1.01_Gm08_9741542_T_C | 9,741,542 | 3.55 | 2.79E-04 |
| 8 | Known | Protein | 9 | K | BARC_1.01_Gm09_4583421_G_A | 4,583,421 | 3.09 | 8.20E-04 |
|  |  |  | 9 | K | BARC_1.01_Gm09_4921120_C_T | 4,921,120 | 4.71 | 1.93E-05 |
|  |  |  | 9 | K | BARC_1.01_Gm09_4950348_T_C | 4,950,348 | 3.06 | 8.62E-04 |
| 9 | New | Protein | 10 | O | BARC_1.01_Gm10_42691772_T_C | 42,691,772 | 3.23 | 5.92E-04 |
| 10 | New |  | 13 | F | BARC_1.01_Gm13_39736564_T_C | 39,736,564 | 3.19 | 6.47E-04 |
|  |  |  | 13 | F | BARC_1.01_Gm13_39741672_G_T | 39,741,672 | 3.14 | 7.19E-04 |
| 11 | Known |  | 15 | E | BARC_1.01_Gm15_12650201_T_C | 12,650,201 | 3.16 | 6.85E-04 |
| 12 | New | Protein | 17 | D2 | BARC_1.01_Gm17_5042611_T_G | 5,042,611 | 3.42 | 3.82E-04 |
| 13 | Known | Protein | 20 | I | BARC_1.01_Gm20_29395999_T_C | 29,395,999 | 3.93 | 1.18E-04 |
|  |  |  | 20 | I | BARC_1.01_Gm20_29512680_A_G | 29,512,680 | 3.69 | 2.03E-04 |
|  |  |  | 20 | I | BARC_1.01_Gm20_29594697_A_G | 29,594,697 | 3.93 | 1.18E-04 |
|  |  |  | 20 | I | BARC_1.01_Gm20_29983050_A_G | 29,983,050 | 3.03 | 9.38E-04 |

^a^  “Known” indicates that the chromosome region corresponded to a previously reported seed oil QTL and “New” indicates a new association identified in this study.

^b^ The word “Protein” indicates either the marker or a marker located in close proximity is also associated with seed protein concentration.
