# Supplementary material for: A genome-wide association study of seed protein and oil content in soybean
Source: BMC Genomics. 2014 Jan 2;15:1. doi: 10.1186/1471-2164-15-1 (PMC3890527; doi:10.1186/1471-2164-15-1)
Supplement: Additional file 4 — Soybean germplasm accessions analyzed in this study. Soybean germplasm accessions [with Plant Introduction (PI) numbers] analyzed in this study and information including country of origin and phenotypic data reported in GRIN. The rightmost four columns display the seed protein and oil values reported in GRIN and those measured in the present study. [file 1471-2164-15-1-S4.doc]

**Additional file 4** **- Soybean germplasm accessions analyzed in this study.**

Soybean germplasm accessions [with Plant Introduction (PI) numbers] analyzed in this study and information including country of origin and phenotypic data reported in GRIN. The rightmost four columns display the seed protein and oil values reported in GRIN and those measured in the present study.

| PI | MG | Origin | Stem Growth | Flow Color | Hilum color | Pod color | Pubescence color | Seed coat color | Seed Size | Protein conc. (%) (GRIN) | Protein conc. (%) (from this study) | Oil conc. (%) (GRIN) | Oil conc. (%) (from this study) |
| --- | --- | --- | --- | --- | --- | --- | --- | --- | --- | --- | --- | --- | --- |
| FC19976 | II | JA | DET | WH | YE | TAN | GR | YE | 22.8 | 46.3 | 45.86 | 16.2 | 15.41 |
| PI157398 | IV | KO | DET | PU | BR | BR | TW | YE | 18.2 | 41.8 | 38.42 | 18.5 | 17.86 |
| PI157405 | IV | KO | DET | PU | GR | BL | GR | GE | 17.0 | 41.7 | 39.82 | 18.7 | 17.82 |
| PI157435 | IV | KO | IND | PU | BR | BR | GR | BR | 9.9 | 41.1 | 39.23 | 18.6 | 15.86 |
| PI157437 | IV | KO | IND | WH | BUFF | BR | GR | YE | 17.6 | 41.7 | 38.88 | 21.6 | 19.55 |
| PI157453 | IV | KO | DET | PU | BR | BL | TW | GE | 15.5 | 41.9 | 41.33 | 19.5 | 17.69 |
| PI157462 | IV | KO | DET | PU | BUFF | BL | GR | GE | 10.5 | 41.7 | 40.82 | 19.5 | 17.18 |
| PI171432 | IV | CH | IND | WH | BUFF | BR | GR | GE | 13.8 | 40.8 | 42.58 | 20.0 | 17.08 |
| PI171450 | III | JA | DET | PU | BR | TAN | TW | YE | 11.0 | 46.3 | 44.50 | 15.3 | 14.64 |
| PI179826 | IV | CH | IND | WH | BR | BR | GR | BR | 22.8 | 40.2 | 38.59 | 21.4 | 19.23 |
| PI187152 | III | JA | DET | PU | BUFF | TAN | TW | YE | 12.9 | 40.2 | 41.28 | 19.7 | 17.31 |
| PI196164 | III | JA | DET | PU | BL | BL | TW | BL | 8.6 | 40.6 | 40.61 | 19.6 | 18.08 |
| PI200482 | II | JA | DET | PU | BUFF | BR | GR | YE | 12.3 | 40.6 | 43.84 | 21.1 | 14.85 |
| PI200485 | III | JA | DET | PU | BUFF | TAN | TW | YE | 13.9 | 40.7 | 40.56 | 19.0 | 17.23 |
| PI200552 | II | JA | DET | WH | BR | TAN | TW | YE | 11.8 | 41.2 | 44.84 | 15.1 | 14.57 |
| PI200596 | II | CH | IND | PU | YE | TAN | GR | YE | 18.4 | 41.5 | 43.51 | 19.4 | 17.58 |
| PI219787 | IV | JA | DET | PU | BR | BR | TW | YE | 16.8 | 40.2 | 38.04 | 19.0 | 17.55 |
| PI227321 | II | JA | IND | PU | YE | TAN | GR | YE | 17.5 | 40.8 | 42.02 | 19.1 | 18.49 |
| PI227684 | II | JA | DET | WH | BR | BR | TW | GE | 12.2 | 40.3 | 43.38 | 17.5 | 16.05 |
| PI229333 | III | JA | DET | PU | BUFF | TAN | TW | YE | 13.4 | 40.5 | 40.61 | 19.5 | 17.52 |
| PI229336 | III | JA | DET | PU | BUFF | TAN | TW | YE | 14.1 | 40.9 | 39.50 | 18.7 | 18.01 |
| PI229349 | IV | JA | DET | PU | BR | BR | TW | YE | 19.1 | 40.3 | 36.49 | 18.9 | 17.40 |
| PI243522 | IV | JA | DET | PU | BR | BR | TW | YE | 17.2 | 40.5 | 35.48 | 17.6 | 18.60 |
| PI243532 | III | JA | DET | WH | BR | BR | TW | YE | 12.7 | 47.8 | 44.58 | 16.2 | 15.89 |
| PI246365 | IV | JA | DET | WH | YE | BR | GR | YE | 20.8 | 40.4 | 38.01 | 17.5 | 15.28 |
| PI248513 | IV | JA | DET | WH | YE | BR | TW | YE | 18.3 | 40.6 | 38.57 | 17.5 | 16.08 |
| PI248515 | IV | JA | DET | WH | YE | BR | GR | YE | 21.5 | 40.3 | 38.08 | 17.4 | 15.33 |
| PI253653B | IV | CH | DET | PU | YE | BR | GR | YE | 21.5 | 42.1 | 43.47 | 19.5 | 17.32 |
| PI253654 | IV | CH | IND | WH | BUFF | BR | GR | YE | 11.3 | 46.6 | 49.07 | 17.2 | 14.73 |
| PI253656B | IV | CH | IND | PU | BR | BL | TW | BR | 9.6 | 46.5 | 46.10 | 15.4 | 13.61 |
| PI253663 | IV | CH | IND | WH | BUFF | BR | GR | YE | 10.8 | 46.6 | 46.04 | 16.5 | 15.32 |
| PI253665C | IV | CH | DET | PU | BUFF | BR | GR | YE | 17.7 | 40.5 | 41.22 | 20.0 | 16.47 |
| PI253666A | IV | CH | IND | WH | BUFF | BR | GR | YE | 12.0 | 47.9 | 49.38 | 15.7 | 14.24 |
| PI266807D | IV | CH | IND | PU | BUFF | BR | GR | YE | 13.8 | 41.3 | 40.88 | 19.9 | 17.46 |
| PI291286 | II | CH | IND | PU | BR | BR | TW | YE | 14.5 | 46.9 | 43.54 | 17.8 | 17.49 |
| PI291288 | II | CH | IND | PU | BR | BR | TW | YE | 14.4 | 48.7 | 42.37 | 17.4 | 17.43 |
| PI291302B | II | CH | IND | WH | BUFF | BR | GR | YE | 17.1 | 46.0 | 44.30 | 18.1 | 17.74 |
| PI291302C | II | CH | IND | PU | YE | BR | GR | YE | 15.1 | 46.8 | 42.58 | 17.6 | 17.24 |
| PI291306B | III | CH | IND | PU | YE | BR | GR | YE | 18.6 | 46.4 | 40.20 | 18.6 | 18.08 |
| PI291309B | II | CH | IND | WH | BL | BR | TW | BR | 18.1 | 48.4 | 44.87 | 18.5 | 16.83 |
| PI291310C | II | CH | IND | WH | BR | BR | TW | YE | 18.1 | 47.2 | 40.87 | 18.1 | 17.57 |
| PI297528 | II | CH | IND | WH | BUFF | BR | GR | YE | 15.7 | 46.9 | 43.33 | 18.1 | 18.45 |
| PI339995 | III | KO | DET | PU | BUFF | BR | GR | YE | 16.6 | 47.7 | 43.18 | 18.0 | 17.23 |
| PI360843 | III | JA | IND | WH | YE | BR | GR | YE | 19.9 | 48.4 | 43.28 | 18.4 | 17.17 |
| PI361053 | II | CH | IND | PU | YE | TAN | GR | YE | 19.3 | 46.8 | 43.33 | 18.7 | 17.41 |
| PI361101 | III | KO | IND | WH | YE | BR | GR | YE | 16.7 | 46.0 | 38.09 | 18.0 | 18.83 |
| PI385942 | IV | JA | DET | PU | YE | BR | GR | YE | 27.3 | 46.4 | 41.23 | 15.1 | 16.13 |
| PI391585 | II | CH | IND | WH | BUFF | BR | GR | YE | 17.2 | 47.7 | 43.54 | 18.1 | 18.00 |
| PI393538 | III | JA | DET | WH | BR | TAN | TW | YE | 10.9 | 46.3 | 42.57 | 16.0 | 17.79 |
| PI393540 | IV | JA | DET | WH | BR | TAN | TW | YE | 11.3 | 46.0 | 43.86 | 15.9 | 16.19 |
| PI398296 | II | KO | DET | PU | BUFF | BR | GR | YE | 12.8 | 46.7 | 41.92 | 17.8 | 17.51 |
| PI398299 | IV | KO | DET | PU | BUFF | BR | GR | GE | 7.7 | 48.5 | 45.30 | 13.2 | 14.67 |
| PI398446 | IV | KO | IND | PU | BL | TAN | TW | BL | 11.1 | 47.4 | 40.20 | 17.2 | 16.79 |
| PI398620 | III | KO | DET | PU | BUFF | TAN | GR | YE | 16.9 | 48.0 | 44.46 | 18.4 | 17.27 |
| PI398705 | IV | KO | IND | PU | BL | TAN | TW | YE | 9.7 | 46.2 | 46.22 | 15.5 | 14.84 |
| PI398706 | IV | KO | DET | WH | BL | BR | TW | YE | 10.7 | 47.9 | 42.46 | 16.2 | 15.94 |
| PI398830 | IV | KO | DET | PU | BR | BR | TW | YE | 17.9 | 46.2 | 42.37 | 17.9 | 16.79 |
| PI404173A | IV | CH | IND | WH | BUFF | TAN | GR | YE | 16.0 | 46.2 | 43.95 | 17.3 | 16.61 |
| PI404174 | IV | CH | IND | WH | BUFF | TAN | GR | YE | 15.8 | 45.9 | 43.32 | 17.0 | 16.49 |
| PI404177 | IV | CH | IND | PU | YE | BR | GR | YE | 14.9 | 51.4 | 45.76 | 15.5 | 14.66 |
| PI404183 | IV | CH | DET | WH | BUFF | TAN | GR | YE | 12.1 | 46.2 | 41.32 | 17.5 | 17.54 |
| PI404186 | III | CH | DET | PU | BL | BR | GR | YE | 13.4 | 45.9 | 40.12 | 16.8 | 16.67 |
| PI404196B | III | CH | IND | PU | BUFF | TAN | GR | YE | 9.4 | 47.2 | 43.28 | 16.7 | 16.35 |
| PI407655B | II | CH | IND | PU | BL | BR | TW | GR | 14.2 | 47.6 | 42.55 | 17.6 | 17.39 |
| PI407658A | IV | CH | DET | WH | BL | TAN | TW | YE | 14.6 | 47.8 | 41.96 | 16.5 | 16.67 |
| PI407658B | IV | CH | IND | WH | GR | BR | GR | GR | 14.3 | 43.3 | 42.77 | 16.5 | 16.21 |
| PI407658C | IV | CH | DET | WH | BL | TAN | TW | YE | 14.6 | 47.2 | 45.64 | 17.1 | 15.74 |
| PI407717 | II | CH | IND | WH | BUFF | BR | GR | YE | 18.9 | 47.8 | 42.47 | 19.7 | 18.23 |
| PI407719 | II | CH | IND | WH | BUFF | BR | GR | YE | 18.3 | 47.0 | 41.45 | 19.2 | 18.63 |
| PI407729 | IV | CH | IND | WH | BL | BR | TW | BL | 9.0 | 46.9 | 40.73 | 16.2 | 15.36 |
| PI407731 | IV | CH | DET | WH | BUFF | BR | GR | YE | 19.3 | 46.2 | 43.93 | 17.2 | 15.92 |
| PI407736 | IV | CH | IND | WH | BUFF | BR | GR | YE | 19.2 | 47.5 | 41.86 | 16.8 | 16.00 |
| PI407773A | IV | KO | DET | WH | BL | BR | TW | YE | 9.8 | 47.5 | 43.01 | 16.5 | 15.63 |
| PI407796 | IV | KO | DET | PU | BL | TAN | TW | BL | 10.1 | 46.5 | 42.58 | 16.1 | 15.13 |
| PI407805A | IV | KO | DET | PU | BUFF | TAN | GR | YE | 7.6 | 47.0 | 41.70 | 15.9 | 16.01 |
| PI407810 | III | KO | IND | PU | BL | TAN | TW | BL | 11.0 | 46.9 | 40.50 | 17.3 | 17.45 |
| PI407914C | IV | KO | IND | PU | BR | BR | TW | YE | 12.4 | 47.1 | 43.59 | 14.9 | 15.51 |
| PI407918B | IV | KO | DET | PU | BR | TAN | TW | YE | 12.3 | 46.9 | 43.44 | 15.0 | 16.02 |
| PI407947 | IV | KO | IND | PU | YE | BR | GR | YE | 19.4 | 46.4 | 43.34 | 16.6 | 16.74 |
| PI408020C | IV | KO | DET | WH | BUFF | BR | GR | YE | 14.8 | 41.7 | 39.84 | 16.6 | 16.45 |
| PI408083A | IV | KO | DET | WH | BR | TAN | TW | GE | 10.2 | 46.2 | 43.96 | 15.5 | 15.84 |
| PI408097 | IV | KO | DET | PU | BR | BR | TW | BR | 13.3 | 46.2 | 44.18 | 15.5 | 15.26 |
| PI408125A | IV | KO | DET | WH | BUFF | TAN | GR | YE | 21.1 | 46.1 | 42.68 | 16.8 | 16.88 |
| PI408200A | IV | KO | DET | WH | BUFF | TAN | GR | YE | 15.4 | 47.0 | 43.35 | 17.5 | 16.36 |
| PI408201A | IV | KO | DET | PU | BR | BR | TW | YE | 12.3 | 46.3 | 42.53 | 15.3 | 15.13 |
| PI408280 | IV | KO | DET | PU | YE | TAN | GR | YE | 10.2 | 46.5 | 42.64 | 16.9 | 16.73 |
| PI408287 | IV | KO | DET | PU | YE | TAN | GR | YE | 7.9 | 48.0 | 45.23 | 15.6 | 15.10 |
| PI416749 | II | JA | DET | PU | BUFF | BR | GR | YE | 20.3 | 46.4 | 38.85 | 16.8 | 18.58 |
| PI416750 | III | JA | IND | WH | YE | BR | GR | YE | 20.8 | 47.7 | 42.14 | 18.1 | 16.96 |
| PI416773 | II | JA | DET | PU | BR | TAN | TW | YE | 9.5 | 48.0 | 43.60 | 15.2 | 13.95 |
| PI416857 | IV | JA | DET | PU | BR | BR | TW | YE | 19.0 | 46.1 | 41.98 | 17.1 | 16.65 |
| PI416868A | III | JA | IND | WH | BL | TAN | TW | YE | 11.4 | 47.8 | 44.77 | 16.2 | 15.77 |
| PI416868B | III | JA | DET | WH | BL | TAN | GR | YE | 11.8 | 47.4 | 44.45 | 16.3 | 16.21 |
| PI416904A | II | CH | IND | PU | YE | TAN | GR | YE | 18.6 | 47.1 | 42.68 | 18.9 | 17.91 |
| PI416941 | II | JA | IND | PU | YE | BR | GR | YE | 19.6 | 47.6 | 43.50 | 18.2 | 16.88 |
| PI416983 | IV | JA | DET | WH | BUFF | BR | GR | YE | 13.3 | 46.0 | 40.60 | 18.1 | 18.68 |
| PI416986 | II | JA | DET | PU | YE | TAN | TW | YE | 14.2 | 48.8 | 43.85 | 16.6 | 15.72 |
| PI417022 | IV | JA | DET | WH | BUFF | BR | GR | YE | 9.1 | 46.7 | 47.10 | 16.5 | 15.64 |
| PI417029 | II | JA | IND | PU | YE | TAN | GR | YE | 18.2 | 48.4 | 44.85 | 17.6 | 16.27 |
| PI417040B | II | CH | IND | WH | BUFF | BR | GR | YE | 21.1 | 47.9 | 42.50 | 17.2 | 17.41 |
| PI417066 | III | JA | DET | PU | BUFF | TAN | TW | YE | 17.3 | 46.4 | 45.38 | 17.4 | 15.30 |
| PI417096 | IV | JA | DET | PU | BL | BL | TW | BL | 10.5 | 46.0 | 41.40 | 17.3 | 16.99 |
| PI417100 | III | JA | IND | WH | BL | BR | TW | BL | 17.8 | 46.7 | 41.02 | 19.3 | NA |
| PI417135A | IV | JA | IND | PU | BL | BR | TW | YE | 18.1 | 46.1 | 42.90 | 14.6 | 14.14 |
| PI417151 | II | JA | DET | WH | BUFF | TAN | GR | YE | 11.9 | 47.6 | 43.44 | 15.7 | 15.11 |
| PI417174 | II | JA | DET | WH | YE | BR | GR | YE | 18.5 | 47.9 | 42.23 | 18.7 | 17.91 |
| PI417176 | IV | JA | DET | WH | YE | BR | GR | YE | 20.2 | 46.8 | 41.22 | 17.5 | 16.61 |
| PI417217 | IV | JA | IND | WH | BUFF | BR | GR | YE | 16.4 | 40.5 | 37.63 | 19.5 | 19.70 |
| PI417243 | IV | CH | IND | WH | BL | TAN | TW | BL | 12.3 | 46.5 | 42.96 | 16.8 | 16.02 |
| PI417244 | III | JA | DET | PU | BUFF | BR | GR | GE | 12.7 | 46.3 | 40.45 | 18.1 | 17.70 |
| PI417248 | III | JA | DET | PU | BR | TAN | TW | YE | 11.4 | 46.5 | 42.72 | 17.2 | 15.45 |
| PI417249 | IV | JA | DET | WH | BR | BR | TW | YE | 15.2 | 47.2 | 39.99 | 16.9 | 17.11 |
| PI417254 | IV | JA | DET | PU | BUFF | TAN | GR | YE | 15.5 | 45.9 | 40.26 | 17.0 | 16.60 |
| PI417268 | II | JA | DET | WH | BR | TAN | TW | YE | 14.4 | 46.5 | 41.67 | 17.8 | 17.48 |
| PI417291 | III | JA | IND | WH | BUFF | BR | GR | YE | 20.2 | 47.5 | 42.54 | 18.7 | 17.99 |
| PI417298 | IV | JA | DET | PU | BUFF | TAN | GR | YE | 10.9 | 46.8 | 41.80 | 17.5 | 16.91 |
| PI417304 | II | JA | DET | WH | BUFF | TAN | GR | YE | 14.1 | 48.3 | 44.86 | 14.9 | 13.89 |
| PI417309A | III | JA | DET | PU | BR | BR | TW | GE | 17.4 | 47.5 | 41.53 | 17.3 | 18.32 |
| PI417328 | III | JA | DET | WH | BUFF | TAN | GR | YE | 12.8 | 47.8 | 43.37 | 15.9 | 15.58 |
| PI417349 | II | JA | IND | PU | YE | TAN | GR | YE | 18.9 | 46.7 | 42.26 | 18.0 | 17.74 |
| PI417382 | IV | CH | DET | PU | BL | BR | GR | GE | 25.8 | 46.7 | 44.88 | 17.6 | 15.84 |
| PI417452 | II | JA | DET | WH | BR | BR | TW | YE | 12.3 | 46.9 | 43.38 | 16.3 | 15.68 |
| PI417482 | III | JA | DET | PU | BR | BR | TW | BR | 22.6 | 46.2 | 44.86 | 17.6 | 16.05 |
| PI417485 | III | JA | DET | WH | BR | BR | TW | YE | 22.7 | 41.4 | 38.81 | 18.9 | 17.49 |
| PI417487 | II | JA | DET | WH | BUFF | TAN | GR | YE | 12.3 | 48.2 | 43.95 | 15.5 | 15.09 |
| PI423833A | IV | KO | IND | WH | BL | TAN | TW | GE | 9.8 | 47.5 | 46.41 | 14.6 | 14.58 |
| PI423836 | IV | KO | DET | WH | BL | TAN | TW | GE | 9.7 | 46.2 | 46.80 | 15.7 | 14.43 |
| PI423843 | IV | KO | DET | PU | BUFF | TAN | GR | YE | 21.8 | 46.0 | 42.11 | 17.4 | 17.74 |
| PI423850 | IV | KO | IND | PU | YE | TAN | GR | YE | 10.0 | 46.7 | 44.21 | 14.8 | 14.47 |
| PI423877 | IV | JA | IND | PU | BR | TAN | TW | YE | 13.5 | 40.2 | 38.72 | 17.8 | 16.79 |
| PI423902 | IV | JA | DET | WH | BR | TAN | TW | YE | 17.8 | 40.9 | 37.34 | 17.4 | 17.34 |
| PI423932 | II | JA | DET | PU | BR | TAN | TW | YE | 10.0 | 46.7 | 43.41 | 15.0 | 13.87 |
| PI423979 | IV | JA | DET | WH | BUFF | BR | GR | YE | 13.0 | 47.7 | 42.02 | 16.8 | 15.88 |
| PI424005 | IV | KO | DET | WH | BL | BL | TW | GE | 3.7 | 46.3 | 46.07 | 12.6 | NA |
| PI424201 | II | CH | DET | WH | BUFF | BR | GR | YE | 15.7 | 46.0 | 40.45 | 18.8 | 19.10 |
| PI424258 | IV | KO | DET | PU | BR | TAN | TW | BR | 8.0 | 47.2 | 43.66 | 16.2 | 15.48 |
| PI424275 | IV | KO | IND | WH | BL | BL | TW | GE | 8.4 | 46.1 | 46.04 | 14.9 | 14.41 |
| PI424321 | IV | KO | IND | WH | BL | BL | TW | GE | 8.3 | 46.0 | 45.97 | 14.8 | 14.31 |
| PI424367 | IV | KO | DET | WH | BL | BL | TW | GE | 8.3 | 46.3 | 45.56 | 14.6 | 14.44 |
| PI424581 | IV | KO | DET | PU | BR | BR | TW | BR | 27.5 | 41.7 | 41.63 | 16.8 | 16.06 |
| PI424583 | IV | KO | DET | WH | BL | TAN | TW | GE | 9.3 | 47.6 | 46.97 | 14.8 | 14.31 |
| PI427088C | II | CH | IND | PU | BR | BR | TW | YE | 14.8 | 47.9 | 41.16 | 18.8 | 18.55 |
| PI430596 | II | CH | DET | WH | BUFF | TAN | GR | YE | 9.7 | 46.6 | 44.83 | 16.6 | 16.57 |
| PI430597 | II | CH | IND | WH | BR | BR | TW | BR | 9.8 | 46.8 | 47.65 | 15.1 | 13.91 |
| PI430598B | IV | CH | IND | PU | BL | BR | TW | GE | 13.4 | 45.9 | 42.03 | 15.8 | 15.98 |
| PI437563 | III | CH | DET | WH | BUFF | BR | GR | YE | 12.5 | 46.7 | 41.15 | 16.6 | 19.23 |
| PI437568 | II | CH | IND | PU | BR | BR | TW | BR | 13.7 | 41.9 | 41.15 | 20.4 | 18.37 |
| PI437572 | II | CH | IND | PU | BL | BR | TW | BL | 11.9 | 46.8 | 42.30 | 15.7 | 15.03 |
| PI437592 | II | CH | IND | WH | BUFF | BR | GR | YE | 18.7 | 41.3 | 38.10 | 23.1 | 21.93 |
| PI437647 | II | CH | IND | PU | BUFF | BR | GR | YE | 17.3 | 41.0 | 40.81 | 20.9 | 19.21 |
| PI437685B | II | CH | DET | PU | BL | TAN | GR | YE | 14.1 | 46.1 | 43.75 | 16.2 | 16.02 |
| PI437698 | II | CH | IND | WH | BUFF | BR | GR | YE | 14.6 | 41.4 | 39.57 | 20.7 | 19.95 |
| PI437699 | II | CH | IND | WH | YE | BR | GR | YE | 22.0 | 41.4 | 42.10 | 21.3 | 18.68 |
| PI437711A | II | CH | IND | PU | BR | BR | TW | YE | 12.8 | 47.0 | 43.75 | 16.6 | 16.37 |
| PI437711B | IV | CH | IND | PU | BR | BR | TW | YE | 12.8 | 47.6 | 45.01 | 15.6 | 15.76 |
| PI437715 | II | CH | IND | WH | BUFF | BR | GR | YE | 14.9 | 40.8 | 39.88 | 21.3 | 18.26 |
| PI437718 | II | CH | IND | PU | BL | BR | TW | BL | 11.8 | 47.2 | 43.39 | 16.1 | 14.78 |
| PI437722 | II | CH | IND | WH | BUFF | BR | GR | YE | 22.8 | 40.7 | 43.07 | 21.9 | 17.78 |
| PI437743 | II | CH | DET | WH | BUFF | BL | GR | YE | 12.4 | 46.4 | 42.43 | 16.9 | 16.99 |
| PI437749 | IV | CH | DET | PU | BL | BR | TW | GE | 22.1 | 46.6 | 43.43 | 15.5 | 16.28 |
| PI437770 | III | CH | IND | WH | BL | BR | GR | BL | 11.0 | 46.3 | 43.12 | 15.8 | 15.58 |
| PI437845B | II | CH | IND | PU | YE | BR | GR | YE | 15.6 | 47.0 | 41.25 | 18.4 | 18.07 |
| PI437845D | IV | CH | DET | PU | YE | BR | GR | YE | 13.3 | 46.3 | 42.54 | 16.4 | 16.49 |
| PI437873 | II | CH | IND | WH | YE | BR | GR | YE | 21.1 | 40.7 | 38.67 | 21.6 | 19.35 |
| PI437877B | II | CH | IND | WH | YE | TAN | GR | YE | 21.7 | 47.5 | 45.80 | 17.7 | 16.08 |
| PI437882A | II | CH | IND | PU | YE | BR | GR | YE | 16.7 | 46.9 | 44.10 | 17.1 | 16.72 |
| PI437890B | II | CH | IND | PU | BR | BR | TW | YE | 13.6 | 46.8 | 44.40 | 18.4 | 17.52 |
| PI437899 | II | CH | IND | PU | BR | BR | TW | YE | 15.5 | 46.9 | 42.53 | 18.4 | 17.70 |
| PI437902C | II | CH | IND | WH | BR | BR | TW | YE | 15.5 | 46.6 | 43.64 | 18.4 | 17.55 |
| PI437904 | II | CH | IND | PU | BL | BR | TW | YE | 13.0 | 41.0 | 37.95 | 18.0 | 18.60 |
| PI437908 | II | CH | IND | PU | BL | BR | TW | YE | 14.1 | 41.4 | 37.54 | 20.3 | 19.23 |
| PI437916 | IV | CH | IND | PU | BL | TAN | TW | BL | 11.7 | 48.9 | 46.27 | 14.4 | 14.82 |
| PI438070 | II | CH | IND | WH | BUFF | TAN | TW | YE | 16.4 | 46.1 | 41.31 | 19.2 | 20.79 |
| PI438144 | II | CH | IND | PU | YE | BR | GR | YE | 19.9 | 47.4 | 45.53 | 17.5 | 15.74 |
| PI438226 | II | CH | IND | PU | BR | BR | TW | YE | 17.6 | 47.1 | 43.05 | 17.5 | 17.36 |
| PI438304B | IV | KO | DET | PU | BL | TAN | TW | BL | 8.6 | 47.7 | 43.38 | 14.2 | 14.67 |
| PI445844 | IV | CH | DET | WH | BUFF | TAN | GR | YE | 18.0 | 46.1 | 41.39 | 14.6 | 16.62 |
| PI445845 | III | CH | DET | WH | BUFF | TAN | GR | YE | 16.9 | 50.4 | 50.55 | 13.2 | 13.12 |
| PI446893 | IV | CH | IND | PU | BUFF | BR | GR | YE | 13.4 | 40.4 | 41.42 | 19.0 | 17.79 |
| PI458184 | IV | KO | DET | PU | BL | TAN | TW | BL | 7.0 | 47.5 | 46.46 | 14.0 | 12.84 |
| PI458226 | IV | KO | DET | WH | BL | TAN | TW | GE | 9.6 | 48.0 | 47.18 | 15.4 | 14.29 |
| PI458227 | IV | KO | IND | PU | BUFF | TAN | GR | YE | 7.5 | 46.1 | 43.01 | 16.6 | 15.74 |
| PI458282 | IV | KO | DET | PU | BUFF | BR | GR | YE | 10.0 | 41.3 | 41.72 | 18.1 | 16.31 |
| PI464920B | III | CH | DET | WH | BUFF | BR | GR | YE | 17.4 | 40.0 | 41.39 | 19.8 | 19.03 |
| PI464922 | II | CH | IND | PU | GR | TAN | GR | YE | 14.0 | 40.7 | 40.98 | 20.8 | 18.71 |
| PI464941 | II | CH | IND | WH | BUFF | BR | GR | YE | 17.7 | 41.0 | 42.11 | 18.1 | 18.30 |
| PI47131 | II | CH | IND | PU | BL | BR | TW | BL | 11.8 | 41.1 | 41.26 | 17.7 | 15.94 |
| PI475785 | III | CH | DET | PU | GR | BR | GR | YE | 17.7 | 40.4 | 39.40 | 20.2 | 18.78 |
| PI483082A | IV | KO | DET | WH | BUFF | BR | GR | GE | 11.2 | 40.9 | 39.47 | 16.3 | 15.79 |
| PI486354A | IV | KO | DET | PU | YE | BR | GR | YE | 21.2 | 40.4 | 38.89 | 18.9 | 18.60 |
| PI490769 | III | CH | DET | WH | BL | BL | TW | BL | 8.4 | 41.5 | 41.11 | 20.9 | 16.07 |
| PI495017B | IV | CH | IND | PU | BUFF | BR | GR | GE | 13.6 | 46.5 | 43.64 | 16.0 | 16.83 |
| PI504812 | IV | KO | DET | PU | BL | TAN | GR | YE | 9.2 | 41.3 | 36.99 | 18.9 | 19.28 |
| PI506572 | III | JA | DET | WH | BR | BR | TW | GE | 13.5 | 46.3 | 47.57 | 16.0 | 14.63 |
| PI506663 | IV | JA | DET | PU | BR | BR | TW | GE | 17.9 | 40.5 | 41.34 | 19.0 | 16.56 |
| PI506681 | IV | JA | DET | PU | BR | BR | TW | YE | 17.8 | 40.3 | 39.93 | 20.3 | 17.54 |
| PI506721 | III | JA | DET | WH | YE | TAN | TW | YE | 16.4 | 41.7 | 40.13 | 18.8 | 17.68 |
| PI506723 | III | JA | DET | WH | YE | TAN | TW | YE | 16.2 | 41.6 | 40.78 | 20.3 | 17.49 |
| PI506787 | III | JA | DET | WH | BR | TAN | TW | YE | 19.3 | 41.5 | 39.64 | 18.4 | 16.90 |
| PI506825 | II | JA | DET | PU | BR | BR | TW | YE | 13.3 | 40.2 | 38.25 | 19.8 | 18.79 |
| PI506872 | III | JA | DET | WH | BR | BR | TW | YE | 17.5 | 41.5 | 42.21 | 18.6 | 15.69 |
| PI506937 | IV | JA | DET | WH | BR | BR | TW | YE | 29.0 | 40.5 | 37.17 | 21.0 | 20.22 |
| PI506942 | II | JA | DET | WH | BUFF | BR | GR | YE | 20.4 | 40.7 | 44.67 | 21.1 | 16.96 |
| PI507021 | IV | JA | DET | PU | BR | BR | TW | YE | 19.4 | 40.5 | 38.41 | 18.6 | 18.00 |
| PI507026 | IV | JA | DET | PU | BR | BR | TW | YE | 14.5 | 40.4 | 41.45 | 18.8 | 17.20 |
| PI507063 | II | JA | DET | WH | GR | BR | TW | YE | 17.9 | 41.8 | 41.50 | 20.4 | 18.30 |
| PI507160 | IV | JA | DET | PU | BL | TAN | GR | YE | 17.4 | 47.2 | 44.33 | 16.1 | 16.64 |
| PI507162 | II | JA | IND | PU | YE | TAN | GR | YE | 16.0 | 41.9 | 41.49 | 19.6 | 18.29 |
| PI507164 | II | JA | DET | WH | BUFF | BR | GR | YE | 15.8 | 41.3 | 41.54 | 19.9 | 18.48 |
| PI507197A | III | JA | DET | PU | BUFF | TAN | GR | YE | 11.5 | 46.0 | 41.36 | 15.0 | 15.22 |
| PI507297 | II | JA | IND | PU | BL | BR | GR | YE | 14.3 | 40.2 | 39.25 | 20.9 | 19.66 |
| PI507312 | IV | JA | DET | PU | BUFF | BL | GR | YE | 6.4 | 46.5 | 42.45 | 15.1 | 16.16 |
| PI507353 | II | JA | DET | WH | YE | BR | GR | YE | 17.8 | 40.7 | 39.50 | 20.5 | 19.83 |
| PI507516 | II | JA | DET | WH | BR | BR | TW | GE | 13.4 | 47.7 | 43.37 | 15.2 | 16.64 |
| PI507552 | II | JA | DET | WH | BR | TAN | TW | YE | 10.4 | 41.2 | 40.99 | 17.7 | 16.18 |
| PI507569 | IV | JA | DET | WH | BUFF | BR | GR | BUFF | 17.7 | 46.5 | 43.07 | 16.1 | 16.38 |
| PI54607 | II | CH | IND | WH | BL | BR | TW | YE | 19.9 | 41.1 | 43.79 | 21.6 | 17.93 |
| PI68454 | II | CH | DET | PU | BL | BR | TW | YE | 16.6 | 40.3 | 39.99 | 21.2 | 19.71 |
| PI68457 | II | CH | DET | WH | BUFF | BR | GR | YE | 17.3 | 41.1 | 39.01 | 21.9 | 19.66 |
| PI68644 | IV | CH | DET | PU | YE | BR | GR | YE | 17.8 | 41.5 | 40.17 | 20.3 | 18.89 |
| PI68694 | II | CH | IND | PU | TAN | BR | TW | YE | 15.5 | 41.8 | 42.15 | 20.3 | 17.52 |
| PI68696 | II | CH | IND | PU | BR | BR | GR | YE | 14.7 | 41.5 | 41.20 | 20.6 | 18.48 |
| PI68709 | II | CH | IND | PU | BR | TAN | TW | YE | 13.7 | 41.1 | 41.29 | 21.2 | 18.76 |
| PI68718 | II | CH | IND | PU | BR | BR | TW | YE | 16.2 | 40.2 | 37.61 | 19.9 | 18.59 |
| PI68748 | II | CH | IND | WH | BUFF | BR | GR | YE | 16.5 | 41.4 | 41.63 | 21.2 | 19.28 |
| PI69500 | II | CH | IND | WH | BR | BR | TW | YE | 14.6 | 40.4 | 42.68 | 18.6 | 16.88 |
| PI69507-1 | IV | CH | IND | PU | BR | TAN | TW | YE | 14.9 | 41.3 | 41.10 | 19.9 | 18.33 |
| PI70197 | II | CH | IND | PU | BR | BR | TW | YE | 16.6 | 40.6 | 44.68 | 19.1 | 16.71 |
| PI70224 | II | CH | IND | WH | BL | BR | TW | YE | 16.9 | 40.5 | 45.58 | 20.3 | 17.66 |
| PI70467 | IV | CH | IND | PU | BL | BR | GR | YE | 13.4 | 41.3 | 40.62 | 20.1 | 18.21 |
| PI71444 | IV | CH | IND | WH | BR | BR | TW | YE | 14.2 | 47.7 | 49.23 | 16.4 | 14.32 |
| PI72227 | IV | CH | DET | WH | BUFF | BR | GR | YE | 16.5 | 47.6 | 45.04 | 17.5 | 16.33 |
| PI72337 | II | CH | IND | PU | YE | TAN | GR | YE | 18.0 | 40.2 | 41.12 | 20.4 | 18.62 |
| PI79696 | IV | CH | IND | WH | BL | BR | TW | GE | 15.1 | 40.6 | 38.67 | 19.3 | 17.97 |
| PI79825-1 | IV | CH | IND | WH | BUFF | BR | GR | YE | 15.6 | 40.6 | 40.02 | 20.2 | 18.42 |
| PI79870-4 | IV | CH | IND | PU | BL | BR | TW | YE | 17.0 | 41.2 | 38.71 | 20.9 | 19.18 |
| PI79870-6 | IV | CH | DET | WH | BR | TAN | TW | YE | 13.8 | 40.2 | 39.12 | 19.4 | 17.89 |
| PI80466-2 | IV | JA | DET | WH | BUFF | TAN | GR | YE | 23.6 | 47.2 | 45.70 | 18.9 | 16.87 |
| PI80470 | III | JA | DET | PU | BR | TAN | TW | GE | 16.9 | 41.1 | 40.19 | 21.7 | 19.55 |
| PI80480 | III | JA | IND | WH | BUFF | BR | GR | YE | 17.0 | 40.6 | 37.10 | 22.3 | 21.38 |
| PI80831 | III | JA | IND | WH | YE | BR | GR | YE | 18.2 | 41.9 | 39.28 | 22.2 | NA |
| PI81761 | III | JA | IND | WH | YE | BL | GR | YE | 18.5 | 41.9 | 38.57 | 21.5 | NA |
| PI82312N | IV | KO | DET | PU | BUFF | BR | GR | YE | 15.1 | 41.7 | 40.94 | 20.7 | 18.71 |
| PI83892 | IV | KO | DET | PU | YE | BR | GR | YE | 11.3 | 40.2 | 39.39 | 18.7 | 16.96 |
| PI84610 | III | KO | IND | PU | BL | BR | TW | BL | 11.1 | 41.8 | 41.76 | 19.1 | 16.49 |
| PI84628 | IV | KO | DET | WH | BUFF | TAN | GR | YE | 15.3 | 40.5 | 39.98 | 19.8 | 17.63 |
| PI84646-2 | IV | KO | IND | PU | BUFF | BR | GR | YE | 15.2 | 40.8 | 40.68 | 19.4 | NA |
| PI84669N | IV | KO | IND | WH | BUFF | BR | GR | YE | 15.0 | 41.4 | 38.92 | 21.0 | 19.32 |
| PI84751 | IV | KO | DET | WH | BL | BR | TW | BL | 7.4 | 40.5 | 41.09 | 18.0 | 15.15 |
| PI84928 | II | KO | DET | PU | YE | TAN | GR | YE | 14.3 | 40.5 | 44.72 | 19.8 | 16.75 |
| PI84965 | II | JA | IND | PU | YE | TAN | GR | YE | 18.5 | 40.7 | 43.06 | 18.6 | 17.58 |
| PI84985 | IV | JA | DET | WH | BUFF | TAN | GR | YE | 16.6 | 40.4 | 36.85 | 20.3 | 19.28 |
| PI86081 | III | JA | DET | PU | BUFF | TAN | TW | YE | 13.0 | 41.1 | 40.66 | 19.1 | 17.63 |
| PI86111 | III | JA | DET | PU | BR | BR | TW | YE | 12.1 | 41.0 | 38.12 | 19.7 | 17.42 |
| PI86114 | III | JA | IND | WH | BL | BR | TW | YE | 16.6 | 41.7 | 40.48 | 20.7 | 18.33 |
| PI86136 | IV | JA | DET | WH | BL | BR | TW | BL | 20.8 | 40.9 | 39.42 | 18.9 | 17.43 |
| PI86443 | II | JA | DET | PU | YE | TAN | GR | YE | 18.0 | 41.0 | 45.71 | 19.4 | 16.77 |
| PI86445 | III | JA | IND | PU | YE | BR | GR | YE | 19.8 | 46.5 | 44.33 | 19.6 | 16.32 |
| PI86456 | III | JA | IND | WH | BUFF | BR | GR | YE | 19.1 | 41.7 | 38.02 | 22.1 | NA |
| PI86457 | III | JA | DET | PU | BUFF | TAN | TW | YE | 13.5 | 40.4 | 40.65 | 19.2 | 17.70 |
| PI86463 | II | JA | DET | PU | BUFF | TAN | GR | YE | 17.5 | 41.0 | 42.74 | 17.0 | 15.97 |
| PI86903-3 | IV | KO | IND | WH | BUFF | TAN | GR | YE | 15.4 | 41.4 | 40.37 | 19.2 | 17.22 |
| PI86904-1 | IV | KO | IND | PU | TAN | BR | TW | YE | 16.7 | 41.0 | 40.13 | 18.5 | 17.11 |
| PI86972-2 | IV | KO | IND | PU | YE | BR | GR | YE | 13.3 | 40.5 | 39.88 | 18.9 | 17.06 |
| PI87011 | IV | KO | DET | PU | BUFF | TAN | GR | YE | 14.1 | 40.4 | 43.18 | 18.9 | 16.28 |
| PI87588 | IV | KO | DET | WH | BUFF | TAN | GR | YE | 16.1 | 41.7 | 42.57 | 20.1 | 17.62 |
| PI87615 | III | KO | DET | WH | BL | BR | TW | YE | 16.2 | 40.8 | 39.17 | 20.6 | 18.93 |
| PI87631-3 | IV | JA | IND | WH | BL | BR | TW | YE | 16.2 | 46.8 | 44.77 | 18.6 | 16.40 |
| PI87634 | III | JA | IND | PU | BR | BR | TW | YE | 15.9 | 41.5 | 40.63 | 20.6 | 19.07 |
| PI88287 | III | CH | IND | PU | BR | BL | TW | BR | 13.7 | 41.9 | 40.77 | 19.0 | 17.41 |
| PI88353 | III | CH | IND | WH | YE | BR | GR | YE | 16.6 | 41.2 | 40.54 | 20.4 | 17.94 |
| PI88442 | II | CH | IND | PU | YE | BR | GR | YE | 18.2 | 41.3 | 46.68 | 20.0 | 17.14 |
| PI88444 | IV | CH | DET | WH | BUFF | BR | GR | YE | 16.2 | 40.3 | 38.98 | 19.7 | 19.40 |
| PI88452 | IV | CH | IND | WH | BUFF | BR | GR | YE | 16.9 | 40.5 | 39.23 | 20.7 | 18.21 |
| PI88499 | IV | CH | DET | WH | BUFF | TAN | GR | YE | 16.8 | 41.3 | 41.73 | 19.5 | 17.96 |
| PI89061-3 | IV | CH | IND | WH | BR | TAN | TW | YE | 16.9 | 40.4 | 40.68 | 21.0 | 18.30 |
| PI89130 | III | KO | IND | WH | YE | BL | GR | YE | 19.7 | 40.6 | 38.92 | 22.4 | NA |
| PI89133 | III | KO | DET | PU | YE | BR | GR | YE | 24.7 | 41.4 | 39.46 | 20.4 | 17.48 |
| PI89154-2 | IV | KO | IND | WH | BR | BR | TW | YE | 14.0 | 40.3 | 40.43 | 20.5 | 17.88 |
| PI89170 | II | CH | IND | PU | BR | BR | TW | YE | 15.6 | 40.3 | 41.50 | 19.9 | 18.35 |
| PI89769 | IV | CH | DET | WH | BUFF | TAN | GR | YE | 17.1 | 40.3 | 43.70 | 20.5 | 16.02 |
| PI90221 | IV | KO | IND | PU | BUFF | BR | GR | YE | 16.3 | 40.9 | 40.69 | 19.9 | 17.06 |
| PI90499-1 | III | CH | IND | PU | BR | BR | TW | YE | 16.0 | 41.5 | 42.49 | 21.5 | 17.41 |
| PI90760 | IV | CH | IND | WH | YE | BR | GR | YE | 19.2 | 40.4 | 39.33 | 20.4 | 18.59 |
| PI90763 | IV | CH | IND | PU | BL | TAN | TW | BL | 9.8 | 40.5 | 41.96 | 18.9 | 14.83 |
| PI91100-4 | IV | CH | IND | WH | BUFF | BR | GR | YE | 17.2 | 40.8 | 38.65 | 20.7 | 19.06 |
| PI91151 | III | CH | IND | WH | BUFF | TAN | GR | YE | 20.4 | 46.5 | 45.23 | 18.7 | 15.82 |
| PI91702 | IV | KO | DET | PU | YE | BR | GR | YE | 23.0 | 41.0 | 41.09 | 19.0 | 17.79 |
| PI91725-4 | III | KO | DET | WH | BUFF | BR | GR | YE | 15.0 | 47.7 | 41.05 | 17.0 | 17.54 |
| PI91730 | III | CH | IND | PU | YE | BR | GR | YE | 15.6 | 41.0 | 41.59 | 20.5 | 17.08 |
| PI92571 | II | CH | IND | WH | YE | BR | GR | YE | 19.4 | 40.4 | 43.27 | 19.7 | 18.43 |
| PI92677 | II | CH | DET | WH | BUFF | BR | GR | YE | 18.7 | 41.2 | 42.40 | 20.5 | 17.45 |
| PI92707-2 | IV | CH | IND | WH | BL | BR | TW | YE | 19.2 | 41.0 | 40.85 | 20.7 | 19.31 |
| PI92713 | IV | CH | IND | WH | BL | BR | TW | YE | 17.9 | 41.1 | 38.93 | 20.4 | 19.00 |
| PI96280 | IV | KO | DET | PU | YE | TAN | GR | YE | 13.2 | 40.5 | 40.40 | 19.4 | 17.64 |
| PI96333 | IV | KO | IND | PU | BL | BL | GR | GE | 16.5 | 41.0 | 38.47 | 20.4 | 19.15 |
| PI96808 | IV | KO | DET | WH | YE | BR | GR | YE | 15.2 | 41.8 | 41.29 | 18.9 | 17.30 |
| PI97155 | IV | KO | DET | WH | BUFF | BR | GR | YE | 16.8 | 41.7 | 41.01 | 18.9 | 16.40 |

MG: maturity group; CH: China; JA: Japan; KR: Korea; DET: determinant; IND: indeterminate; WH: white; PU: purple; YE: yellow; GR: gray; TW: tawny; BL: black; BR: brown; GE: green; NA: not assigned.
